# Global inequalities in the double burden of malnutrition and associations with globalisation: a multilevel analysis of Demographic and Health Surveys from 55 low-income and middle-income countries, 1992–2018

**DOI:** 10.1016/S2214-109X(21)00594-5

**Published:** 2022-02-08

**Authors:** Paraskevi Seferidi, Thomas Hone, Ana Clara Duran, Antonio Bernabe-Ortiz, Christopher Millett

**Affiliations:** a Public Health Policy Evaluation unit, School of Public Health, Imperial College London, London, UK; b Center for Food Studies and Research, University of Campinas, São Paulo, Brazil; c CRONICAS Center of Excellence in Chronic Diseases, Universidad Peruana Cayetano Heredia, Lima, Peru; d National School of Public Health, NOVA University, Lisbon, Portugal

## Abstract

**Background:**

Low-income and middle-income countries (LMICs) face a double burden of malnutrition (DBM), whereby overnutrition and undernutrition coexist within the same individual, household, or population. This analysis investigates global inequalities in household-level DBM, expressed as a stunted child with an overweight mother, and its association with economic, social, and political globalisation across country income and household wealth.

**Methods:**

We pooled anthropometric and demographic data for 1 132 069 children (aged <5 years) and their mothers (aged 15–49 years) from 189 Demographic and Health Surveys in 55 LMICs between 1992 and 2018. These data were combined with country-level data on economic, social, and political globalisation from the Konjunkturforschungsstelle Globalisation Index and gross national income (GNI) from the World Bank. Multivariate associations between DBM and household wealth, GNI, and globalisation and their interactions were tested using multilevel logistic regression models with country and year fixed-effects and robust standard errors clustered by country.

**Findings:**

The probability of DBM was higher among richer households in poorer LMICs and poorer households in richer LMICs. Economic globalisation was associated with higher odds of DBM among the poorest households (odds ratio 1·49, 95% CI 1·20–1·86) compared with the richest households. These associations attenuated as GNI increased. Social globalisation was associated with higher odds of DBM (1·39, 95% CI 1·16–1·65), independently of household wealth or country income. No associations were identified between political globalisation and DBM.

**Interpretation:**

Increases in economic and social globalisation were associated with higher DBM, although the impacts of economic globalisation were mostly realised by the world's poorest. The economic patterning of DBM observed in this study calls for subpopulation-specific double-duty actions, which should further aim to mitigate the potential negative and unequal impacts of globalisation.

**Funding:**

UK Biotechnology and Biological Sciences Research Council.

**Translations:**

For the Spanish and Portuguese translations of the abstract see Supplementary Materials section.

## Introduction

Ending malnutrition in all its forms is among the top priorities of the UN Decade of Action on Nutrition.^1^ Overnutrition (ie, dietary excess, overweight and obesity, and diet-related non-communicable disease) and undernutrition (ie, energy and micronutrient deficiencies, such as stunting, wasting, and anaemia) have been viewed as two distinct issues. However, recognition that overnutrition and undernutrition frequently coexist within individuals, households, and populations, with common underlying drivers and causes, is the basis of the emergent double burden of malnutrition (DBM) concept.^2^ Evidence indicates that the prevalence of DBM is high in low-income and middle-income countries (LMICs). More than one-third of 126 LMICs have high prevalence of both undernutrition (stunting >30%, wasting >15%, female thinness >20%) and overweight (>20%). Prevalence of total household-level DBM ranges between 3% and 35% across 126 LMICs (1999–2017), with stunted child–overweight mother pairs being the most prevalent DBM type (1–24%), followed by overweight mother–wasted child (0·3–10%) and thin mother–overweight child pairs (0·1–4%).^3^

DBM varies across country settings^4^ and socioeconomic groups,^5^ although this heterogeneity has not been fully explored. For example, LMICs in the lowest-income quartile are more likely to face population-level DBM compared with LMICs in middle-income and high-income quartiles, whereas household-level DBM is least prevalent in LMICs with lowest and highest gross domestic product levels.^3^ Moreover, associations between individual-level socioeconomic characteristics and DBM vary considerably between countries. For example, the prevalence of stunted child–overweight mother pairs is associated with deprivation in Guatemala, Colombia, and rural Mexico,^6^, ^7^, ^8^ and with affluence in numerous Asian countries, including Bangladesh, Nepal, and Pakistan.^9^, ^10^, ^11^

**Research in context**

**Evidence before this study**

We searched PubMed, Google Scholar, and Scopus on April 27, 2021, using search terms related to socioeconomic status, such as ”income” and “wealth”; or globalisation, such as ”globalisation”, “foreign direct investment”, and ”trade liberalisation”; and malnutrition, such as ”double burden of malnutrition”, ”stunting”, ”overweight”, and ”BMI”. We had no language or date restrictions. Our searches identified several observational studies that investigate associations between the double burden of malnutrition and socioeconomic status at regional or national levels of low-income and middle-income countries. The direction of the identified associations varied across settings and we found no comprehensive investigation of this heterogeneity at global level. We identified no studies that investigate associations between globalisation measures and the double burden of malnutrition, although some studies have examined associations between globalisation and overnutrition or undernutrition separately. Associations between globalisation and overweight and obesity were not consistent across all research studies, population groups, or model specifications, and evidence on the association between globalisation and underweight were mixed and scarce, with no robust multicountry evidence. Finally, we identified a few studies that further explored associations between globalisation and diet-related outcomes, such as body-mass index and food insecurity, across socioeconomic characteristics, which suggest that identified associations might vary across socioeconomic groups.

**Added value of this study**

To our knowledge, our study is the first robust global analysis of inequalities in the double burden of malnutrition that examines associations with different measures of globalisation. We used individual-level data from about 1·1 million child–mother pairs from 189 Demographic and Health Surveys in 55 low-income and middle-income countries globally to characterise the double burden of malnutrition at the household level. We used robust models with country and year fixed-effects to estimate associations with economic, social, and political globalisation. We identified important new evidence that the probability of the double burden of malnutrition at household level differed across country income and household wealth, with higher probability found in richer households of poorer countries and in poorer households of richer countries. Moreover, we identified that economic globalisation was associated with double burden of malnutrition among the poorest households of poorer countries. Social globalisation was associated with higher odds of double burden of malnutrition across all household wealth index groups and country income levels.

**Implications of all the available evidence**

The double burden of malnutrition is unequally distributed across socioeconomic groups and country income in low-income and middle-income country settings. Economic and social globalisation might exacerbate the double burden of malnutrition, with economic globalisation being particularly harmful to the world's poorest populations. Double-duty policy actions that aim to concurrently address all forms of malnutrition should consider how to best counter the potential negative impacts of globalisation.

The emergence of the DBM has coincided with a time of increasing globalisation and a nutrition transition from traditional towards more globalised food systems. Globalisation describes nations' openness to global collaboration and influence, through international economic activities and trade; exchange of culture, ideas, and information; and collaboration between nations through international organisations.^12^ Although globalisation can improve economic development, it has been associated with income and health inequalities, through its impact on labour markets and fiscal and social policy.^13^, ^14^ Globalisation also substantially impacts food systems, shaping diets and diet-related health outcomes.^15^ In emerging economies that have not yet fully addressed undernutrition issues, globalisation can increase the quantity of available food, while deteriorating its quality through the introduction of new ultraprocessed foods. It can also shift consumer preferences and habits and interfere with domestic policy making. These globalisation implications might slow down progress in reducing undernutrition, while introducing overnutrition drivers. Importantly, associations between globalisation and diet-related outcomes can vary across socioeconomic groups.^16^, ^17^, ^18^

A systematic global analysis of the within-country and between-country variation in DBM and the potential role of globalisation in explaining this variation is missing. We aim to address this evidence gap by investigating the probability of DBM, expressed as stunted child with overweight mother living in the same household, by country income and household wealth, and its association with economic, social, and political globalisation. We performed a multilevel analysis of 1·1 million children from 55 LMICs worldwide between 1992 and 2018 to explore the associations between DBM, country income, and globalisation. We used country and year fixed-effects to account for differences between countries and time trends.

## Methods

### Data

We obtained serial, cross-sectional data from the Demographic and Health Surveys (DHS). The DHS programme conducts nationally representative surveys of girls and women of reproductive age (15–49 years) and their children born in the 5 years before the survey in more than 90 LMICs. We used the R package rdhs (version 0.6.3) to identify, access, and download relevant DHS surveys.^19^ We identified 255 DHS surveys from 76 LMICs, from database inception until Dec 31, 2018, with anthropometry indicators. After relevant exclusions (ie, surveys restricted or non-available; surveys with no information on at least one of the following: mother's age or height, child's height-for-age Z-score, wealth index; and surveys with only one time point; appendix 3 p 3), our final dataset included 189 surveys from 55 countries, containing between two and nine surveys per country in the period 1992–2018. An overview of countries and years included in the sample is presented in the appendix 3 (p 4). After excluding pregnant women, individuals with weight or height missing or extreme values, and children who were not living with their mothers (appendix 3 p 3), our dataset consisted of 1 132 069 child–mother pairs.

### Exposure

We used the Konjunkturforschungsstelle (KOF) Globalisation Index as a measure of globalisation.^12^ KOF is a composite index, which uses 43 variables to measure globalisation at country-level and over time, using time-varying weights when aggregating variables to account for changes in their relevance in describing globalisation over time. KOF is defined across three distinct dimensions: economic, social, and political globalisation. Economic globalisation is further disaggregated into trade and financial globalisation, which measure openness in trade flows and international finance and investment, respectively. Social globalisation includes three subcomponents: interpersonal, informational, and cultural globalisation, which refer to interconnections and exchange of people, information, and cultural values and ideas, respectively. Finally, political globalisation refers to engagement in international organisations and treaties. We used all dimensions of globalisation and their subcomponents to understand potential mechanisms. The latest version of the KOF index further disaggregates each globalisation subcomponent into de-facto and de-jure globalisation. De-facto globalisation describes actual globalisation activities, whereas the de-jure indices refer to the policies and institutions that enable and facilitate globalisation activities. Similarly to previous studies,^18^ we used the de-jure indices of globalisation because they are not confounded by potential weak implementation of globalisation-related policies and are a prerequisite for de-facto globalisation.^12^ KOF takes values of 1–100, with higher values indicating higher levels of globalisation. It is available for 203 countries and territories worldwide between 1970 and 2018. In our sample, data on Economic KOF (Comoros) and Trade KOF (Comoros and the Maldives) were missing and excluded from analyses using these indices. A full description of the KOF indices exists elsewhere.^12^

### Outcome

We defined DBM as a stunted child with an overweight mother living in the same household. A child was considered stunted if they had a height-to-age Z score under 2 SDs below the average Z score according to the WHO's 2006 Child Growth Standards. A woman was considered overweight if she had a body-mass index (BMI) of 25kg/m^2^ or higher. Our outcome was a binary variable with value 1 if a child was both stunted and had an overweight mother and 0 in all other cases. Although other measures of DBM exist, we used stunted child–overweight mother pairs as it is the most prevalent and well studied measure of household-level DBM.^3^

### Covariates

We measured economic affluence at household level using the DHS wealth index. The wealth index is a composite measure of relative economic status estimated using household-level information on asset ownership and access to services from individual questionnaires. Quintiles estimated by the DHS based on the population distribution of wealth index in each survey sample were used. To measure country income, we used annual data of the World Bank's Gross National Income (GNI) per capita indicator, converted to 2020 US$ using the World Bank Atlas method. This indicator is used by the World Bank to classify countries by their income.

We considered several demographic and socioeconomic covariates as potential confounders in our analysis. These were wealth index quintiles, GNI, wealth index quintiles and GNI interaction, child's sex (male *vs* female) and age (in months), mother's age (in years), whether mother was breastfeeding at the time of the survey (yes *vs* no), urban or rural residence, number of children living in the household, mother's marital status (currently married *vs* formerly married *vs* never married), and mother's education (no completed education *vs* completed primary education and above). We also adjusted for urbanisation (percentage of urban population) and female unemployment (percentage of female labour force) using country-level data from the World Bank.

### Statistical analysis

All analyses were weighted using denormalised individual DHS survey weights (appendix 3 p 1), which consider sampling design and non-response rates. We estimated weighted means and SEs or frequencies and weighted percentages for all covariates overall and by DBM status. Differences between groups were tested using Student's *t* test or Pearson's χ^2^ test with Rao-Scott correction.

Inequalities in the prevalence of DBM across household wealth and country income and associations between DBM and globalisation were tested. Multilevel logistic regression with country and year fixed-effects and appropriate interaction terms were used to estimate odds ratios [ORs], and 95% CIs were estimated using robust SEs clustered by country. Using post-regression modelling, average marginal effects were estimated to plot differences in the probability of DBM for child–mother pairs in the richest quintile compared with the poorest quintile and for a 10-unit increase of KOF for each wealth index quintile, at increasing levels of GNI by US$100 intervals between $150 and $4950. More details on model specification and statistical analyses are presented in the appendix 3 (p 1).

We performed several sensitivity analyses (appendix 3 p 2). We first tested consistency of our results across different model specifications, including testing several time specifications, mixed-effects models, and multiway standard error clustering. We then tested non-linearity of KOF and GNI, used different cutoff points for mother overweight in south Asian countries, and performed analyses stratified by age and sex. Moreover, we repeated analyses using non-standardised survey weights. As 24·4% of children in the sample had the same mother, we also tested the assumption that all observations in each cluster are independent by fitting models with mother as the level of observation. We also tested interactions with other socioeconomic characteristics beyond wealth index. Finally, we included as model covariates the prevalence of child stunting and mother overweight estimated by country, year, urban versus rural region, and wealth index quintile group and tested their relative contribution to the model fit.^9^ This sensitivity analysis was done to address concerns that associations with stunted child–overweight mother pairs are only attributed to the underlying trends of overweight and stunting in the population and are not a distinct issue.^20^

Statistical analyses were done using Stata, version 15.

### Role of the funding source

The funder had no role in the study design, data collection, data analysis, data interpretation, or writing of the report.

## Results

Across all country-years, 67 580 (6·0%) of 1 132 069 children in the sample were stunted and had an overweight mother, with within-country probability of DBM increasing over time by 1·04 times per year (95% CI 1·03–1·05) after adjusting for relevant covariates. Those children were more likely to be older, boys, live in an urban area, have a higher household wealth index, and have mothers who are older, less likely to be breastfeeding, and of higher education compared with children without DBM (table 1). GNI in the sample increased and became more variable over the study period (1992–2018; appendix 3 p 5). Average country change of GNI was US$1216. Globalisation indices increased over the study period and ranged between 13 and 95 across different dimensions, countries, and years in the sample (appendix 3 p 5). Changes over the study period in the average country varied between 5·2 units (SD 10·2) for economic, 15·6 units (9·5) for social, and 15·1 units (10·1) for political globalisation.

**Table 1:** Sample characteristics overall and by double burden of malnutrition status

		**Overall (N=1 132 069)**	**No DBM (N=1 064 489)**	**DBM (N=67 580)**	**p values**
Quintiles of wealth index		..	..	..	<0·0001
	Poorest households	280 658 (22·9%)	264 937 (23·2%)	15 721 (17·8%)	..
	Poorer households	248 183 (21·3%)	233 679 (21·5%)	14 504 (18·9%)	..
	Middle households	226 224 (20·3%)	212 707 (20·2%)	13 517 (20·9%)	..
	Richer households	202 716 (19·2%)	190 149 (19·0%)	12 567 (21·9%)	..
	Richest households	174 288 (16·3%)	163 017 (16·1%)	11 271 (20·5%)	..
Mean age of mother (SE), years		27·9 (0·01)	27·8 (0·01)	30·0 (0·04)	<0·0001
Mean age of child (SE), months		28·3 (0·03)	28·1 (0·03)	31·7 (0·11)	<0·0001
Mean number of children in the household (SE)		1·89 (0·00)	1·89 (0·00)	1·91 (0·01)	0·0084
Sex of child		..	..	..	<0·0001
	Female	555 530 (48·7%)	524 270 (48·9%)	31 260 (46·0%)	..
	Male	576 539 (51·3%)	540 219 (51·1%)	36 320 (54·0%)	..
Breastfeeding mother		728 599 (66·1%)	692 316 (67·0%)	36 283 (51·9%)	<0·0001
Type of region		..	..	..	<0·0001
	Rural	755 493 (70·1%)	715 512 (70·8%)	39 981 (58·3%)	..
	Urban	376 576 (29·9%)	348 977 (29·2%)	27 599 (41·7%)	..
Marital status		..	..	..	<0·0001
	Currently married	1 042 497 (94·9%)	979 863 (94·9%)	62 634 (95·3%)	..
	Formerly married	57 642 (3·6%)	54 106 (3·6%)	3536 (3·6%)	..
	Never married	31 919 (1·5%)	30 510 (1·6%)	1409 (1·1%)	..
	Missing (%)	11 (<1%)	10 (<1%)	1 (<1%)	..
Mother's education		..	..	..	<0·0001
	No completed education	585 187 (51·1%)	551 217 (51·5%)	33 970 (44·4%)	..
	Completed primary education and above	542 618 (48·8%)	509 339 (48·4%)	33 279 (55·5%)	..
	Missing (%)	4264 (0·1%)	3933 (0·1%)	331 (0·2%)	..
	Mean urbanisation (SE), %	35·7 (0·02)	35·4 (0·02)	41·6 (0·08)	<0·0001
	Mean female unemployment (SE), %	7·0 (0·01)	6·8 (0·01)	10·0 (0·05)	<0·0001

Data are n (%) or mean (SE). Frequencies, means, and SEs are weighted using denormalised individual Demographic Health Ssurvey weights. p values are from Student's *t* test and Pearson's χ^2^ tests for the difference between DBM and no DBM. DBM=double burden of malnutrition.

**Figure 1:**
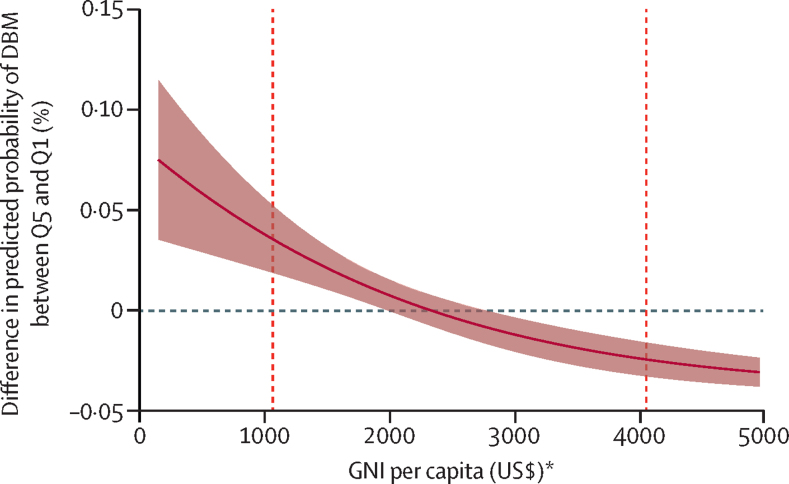
Difference in the probability of the double burden of malnutrition in the richest wealth index quintile compared with the poorest wealth index quintile, at increasing levels of GNI per capita Positive difference (above the horizontal dashed line) indicates that child–mother pairs in the richest wealth index quintile (Q5) have higher probability of DBM than child–mother pairs in the poorest wealth index quintile (Q1). Negative difference (below the horizontal dashed line) indicates that child–mother pairs in the richest wealth index quintile have lower probability of DBM compared with child–mother pairs in the poorest wealth index quintile. Average marginal effects have been calculated at GNI for every US$100 between $150 and $4950. Marginal effects are estimated from a logistic regression between DBM and wealth index quintiles, GNI (per $100), and their interaction, adjusted for country and year fixed-effects, child's sex (male *vs* female) and age (in months), mother's age (in years), whether mother was breastfeeding at the time of the survey (yes *vs* no), urban or rural residence, number of children living in the household, mother's marital status (currently married *vs* formerly married *vs* never married), mother's education (no completed education *vs* completed primary education and above), country-level urbanisation (percentage of urban population), and country-level female unemployment (percentage of female labour force). The shaded area denotes 95% CI clustered by country. Vertical dashed lines indicate the cutoff points for lower-middle-income countries ($1036) and upper-middle-income countries ($4046), as defined by the World Bank in 2021. DBM=double burden of malnutrition. GNI=gross national income. *Calculated with the Atlas method.

The probability of DBM varied significantly across household wealth quintiles with the direction of association decreasing and inverting as GNI increased. The difference in the probability of DBM between the richest and the poorest household wealth quintiles, at GNI between US$150 and $4950 per capita, is shown in figure 1. For countries with low GNI, the difference in the probability of DBM between the richest and the poorest households was positive—ie, the richest child–mother pairs were more likely to have DBM compared with the poorest. For example, for GNI at $150 per capita, the probability of DBM was 7·5% higher (95% CI 3·5–11·5) in the richest quintile compared with the poorest quantile. However, as GNI increased, the direction of the inequality reversed, with the richest child-mother pairs being less likely to have DBM compared with the poorest. For example, for GNI at $4950 per capita, the probability of DBM was 3·1% lower (−3·8% to −2·4) in the richest quintile compared with the poorest quantile.

**Figure 2:**
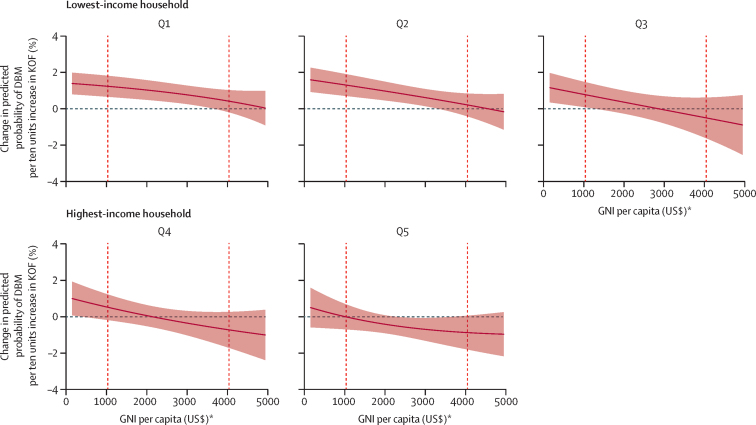
Associations between KOF economic index (per ten units) and DBM across wealth index quintiles (Q1–5), as GNI per capita increases Average marginal effects have been calculated at GNI for every US$100 between $150 and $4950. Vertical dashed lines indicate the cutoff points for lower-middle-income countries ($1036) and upper-middle-income countries ($4046), as defined by the World Bank in 2021. The shaded area denotes 95% CI. DBM=double burden of malnutrition. GNI=gross national income. KOF=Konjunkturforschungsstelle Globalisation Index. *Calculated with the Atlas method.

We observed significant interactions between economic globalisation, household wealth quintile, GNI, and DBM probability (table 2). A ten-unit increase in economic globalisation was associated with 49% higher odds of DBM in the poorest household wealth quintile (OR 1·49; 95% CI 1·20–1·86). However, this association decreased in higher household wealth quintiles and with increasing country GNI per capita. The association between economic globalisation and DBM across all household wealth quintiles, at increasing levels of GNI, is shown in figure 2. The same pattern was observed for the subcomponents of the economic globalisation index, particularly financial globalisation (appendix 3 pp 5–7).

**Table 2:** Associations between the three components of the KOF Globalisation Index (per 10 units) and the double burden of malnutrition (stunted child with overweight mother) and interactions between KOF and wealth index quintile and GNI per capita

		**Economic KOF (n=1 124 245)**		**Social KOF (n=1 127 155)**		**Political KOF (n=1 127 155)**	
		OR (95% CI)	p value	OR (95% CI)	p value	OR (95% CI)	p value
KOF		1·49 (1·20–1·86)	<0·0001	1·39 (1·16–1·65)	<0·0001	0·88 (0·68–1·13)	0·31
GNI		1·05 (1·01–1·10)	0·018	1·00 (0·94–1·07)	0·93	0·98 (0·92–1·03)	0·37
Interactions between KOF and wealth index quintiles							
	Poorest × KOF	1·00 (ref)	..	1·00 (ref)	..	1·00 (ref)	..
	Poorer × KOF	0·96 (0·89–1·04)	0·33	1·06 (0·99–1·13)	0·10	1·14 (1·05–1·22)	<0·0001
	Middle × KOF	0·84 (0·71–0·99)	0·037	1·06 (0·91–1·23)	0·45	1·16 (0·96–1·41)	0·13
	Richer × KOF	0·80 (0·68–0·93)	<0·0001	1·10 (0·93–1·29)	0·27	1·18 (0·92–1·51)	0·20
	Richest × KOF	0·73 (0·64–0·83)	<0·0001	1·10 (0·91–1·33)	0·31	1·15 (0·80–1·60)	0·42
Interaction between KOF and GNI							
	GNI × KOF	0·99 (0·99–1·00)	0·012	1·00 (0·99–1·01	0·98	1·00 (1·00–1·01)	0·36

Models are adjusted for for country and year fixed-effects, and wealth index quintiles, GNI, wealth index quintiles and GNI interaction, child's sex (male *vs* female) and age (in months), mother's age (in years), whether mother was breastfeeding at the time of the survey (yes *vs* no), urban or rural residence, number of children living in the household, mother's marital status (currently married *vs* formerly married *vs* never married), mother's education (no completed education *vs* completed primary education and above), country-level urbanisation (percentage of urban population), and country-level female unemployment (percentage of female labour force). 95% CI are clustered by country. DBM=double burden of malnutrition. GNI=gross national income. KOF=Konjunkturforschungsstelle Globalisation Index. OR=odds ratio.

**Figure 3:**
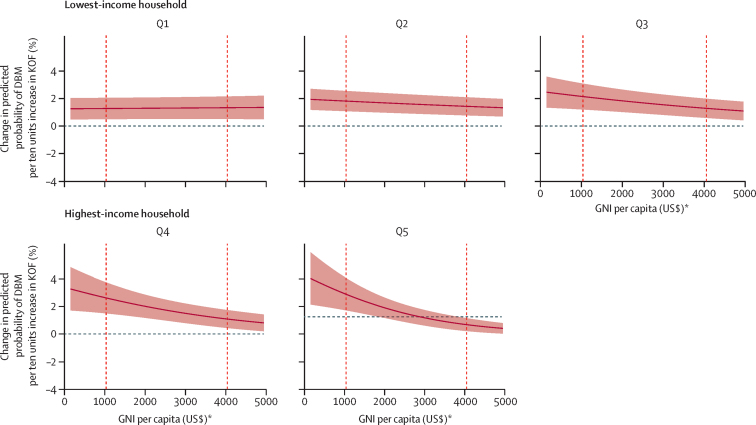
Associations between KOF social index (per ten units) and the DBM across wealth index quintiles (Q1–5), as GNI per capita increases Average marginal effects have been calculated at GNI for every $US100 between $150 and $4950. Vertical dashed lines indicate the cut-off points for lower-middle-income countries ($1036) and upper-middle-income countries ($4046), as defined by the World Bank in 2021. The shaded area denotes 95% CI. DBM=Double burden of malnutrition. GNI=Gross National Income. KOF=Konjunkturforschungsstelle Globalisation Index. *Calculated with the Atlas method.

Social globalisation was also associated with higher odds of DBM, independently of household wealth quintile and GNI (table 2). A ten-unit increase in social globalisation was associated with a 39% increase in the odds of DBM in the lowest household wealth quintile (OR 1·39, 95% CI 1·16–1·65). Increases in GNI attenuated the association between social globalisation and DBM in richer wealth index quintiles, although associations remained significant (figure 3). These associations were driven by the different social globalisation subcomponents, with interpersonal globalisation mainly driving associations among the poorest households, and informational and cultural globalisation driving associations among the richer households of lower-income countries (appendix 3 pp 8–11). Finally, we identified no associations between political globalisation and DBM (table 2).

Our models were generally robust to sensitivity analysis checks. Our results did not change under different time specifications, although associations with social globalisation were weaker and became non-significant when linear time trends for each region or country were considered (appendix 3 p 12). Models with random intercepts and random slopes by region were also consistent with our initial findings (appendix 3 p 13). Clustering standard errors by country and wealth index quintiles or urban versus rural region or level of mother's education or region also did not change our results (appendix 3 p 14). Adding quadratic GNI and KOF in the model did not change estimated coefficients, although models with a quadratic KOF term widened confidence intervals to being non-significant. As none of the KOF quadratic terms were significant, we consider that this model had a poorer fit than our main analysis (appendix 3 p 15). Stratified models (appendix 3 p 16) showed no considerable differences between age and sex groups, whereas models with different overweight cutoff points for South Asian countries provided results similar to our main analysis (appendix 3 p 17). Models weighted using normalised weights (appendix 3 p 17), different specifications of the model at mother-level, and interactions with other socioeconomic determinants (appendix 3 p 18) were also consistent with our initial findings. Finally, adding child stunting and mother overweight prevalence as covariates to the models did not substantially change associations between globalisation and DBM, whereas the contribution of these variables to the model fit were below 9% and 30%, respectively (appendix 3 p 19).

## Discussion

This analysis indicates that household-level DBM is distributed unequally across quintiles of relative wealth in LMICs, and that the direction of this inequality is associated with country income. Specifically, we show that the probability of DBM was higher in the richest households of lower-income LMICs and in the poorest households of higher-income LMICs. Our analysis quantified the relationship between different types of globalisation and DBM, across household wealth and country income. Economic globalisation was associated with higher odds of DBM among the poorest households in the poorest LMICs and this association was attenuated as household wealth and country income increased. Thus, economic globalisation could potentially reduce the gap between the richest and poorest households in lower-income LMICs, but by increasing DBM among the poorest. We also found that social globalisation increased the probability of DBM across all household wealth groups, especially in the richest households of lower-income LMICs.

Previous limited country-level analyses of DBM inequalities have shown consistent findings. For example, analyses in upper-middle-income countries in Latin America have shown that DBM is associated with lower socioeconomic status,^6^, ^7^, ^8^ whereas evidence from mainly lower-middle-income countries in Asia has shown that DBM is associated with higher socioeconomic status.^9^, ^10^, ^11^ No previous analysis has investigated associations between globalisation and DBM. Social globalisation has been associated with higher BMI and prevalence of overweight and obesity.^21^, ^22^, ^23^ Associations between economic and trade globalisation and overnutrition outcomes are less consistent, with some analyses showing significant associations,^24^ whereas others show no or very small associations.^21^, ^22^, ^23^ Finally, a previous analysis has shown that political globalisation was associated with higher odds of overweight among women aged 15–49 years, using DHS data.^23^ However, this analysis did not adjust for interactions with household wealth and country income, and country and year fixed-effects.

Our analysis reinforces previous research showing that globalisation has heterogenous impacts on nutrition-related outcomes across socioeconomic groups. For example, foreign direct investment has been associated with higher BMI among the poorest in rural areas and lower BMI among the richest in urban areas of 38 LMICs.^17^ Similarly, a recent multilevel analysis in 132 countries found a positive association between trade globalisation and food insecurity among households with the lowest income in low-income countries, which was reversed for the wealthiest households of high-income countries.^18^

This study employed a robust methodological approach, using country and year fixed-effect specifications, adjusting for unobserved differences between countries and global underlying trends. Our analysis significantly advances previous work, which has largely used country-level data and ecological analyses, by employing multilevel data from a large sample of 1·1 million children from 55 LMICs. Moreover, we explored interactions with household wealth and country income. Our results remained robust under different sensitivity analyses, including after adjustment for underlying trends of overweight and stunting, indicating that the observed associations with DBM are not solely attributed to underlying child stunting and female overweight trends, but exist as a unique issue. However, our analysis has some limitations. We used data from mothers aged 15–49 years and their children under the age of 5 years to define DBM as a stunted child–overweight mother pair. Although we employed the most common definition of DBM, other manifestations exist and might show diverse associations with globalisation. Future work should further explore these associations to inform the net impact of globalisation on malnutrition in all its forms. We excluded women and children with missing weight and height data (n=535 398 pairs), although sample weights consider non-response. We also excluded women who were pregnant (n=181 225), as BMI is not an appropriate measure of overweight in pregnant women. Pregnant women in our sample were more likely to be younger, have older and female children, live in rural areas, and be poorer. Finally, we used wealth index quintiles to measure household economic status. Wealth index is a relative measure of affluence that is survey specific. We presented within-country associations at increasing levels of country income to allow for more accurate interpretation of wealth index at the global level over time. In a sensitivity analysis, we also used other measures of socioeconomic status—ie, urban versus rural region and mother's education, which showed similar results.

A recent *Lancet* Commission has called for double-duty policy actions that concurrently target the common drivers of all forms of malnutrition.^25^ Our findings indicate that the design of such actions needs to consider and address the potential negative and unequal impacts of globalisation. Although further work is needed to understand the common mechanisms through which globalisation affects DBM, impacts on dietary quality, food environments, and breastfeeding practices can be hypothesised. Trade and financial globalisation can introduce new ultraprocessed foods to local markets,^26^ which can increase overnutrition outcomes and exacerbate food insecurity^18^ by disrupting transition to more diverse nutritious diets and impacting food prices and availability. As further supported by our results, this effect is especially evident in lower-income countries, which still have relatively low market penetration and provide a profitable setting for transnational corporations.^27^ Although economic globalisation can improve women's participation in the workforce, these changes are not always accompanied by proper work conditions, employment security, and paid maternity leave,^28^, ^29^ which are determinants of suboptimal breastfeeding practices.^30^ Social globalisation can also impact people's ideas and perceptions of food and infant care. It can introduce westernised food cultures that are overwhelmed by commercial attitudes towards food, shifting social norms and dietary behaviours, and change food preferences away from traditional diets. It can also increase mass media exposure to infant formula and other breastmilk substitutes that are known barriers to optimum breastfeeding practices.^30^ It is possible that these changes might initially impact affluent groups the most, as shown from our results, given that they are the first to gain access to new infrastructure and technologies facilitated by social globalisation, especially in lower-income countries. Double-duty actions need to be context-specific and subpopulation-specific, given the economic patterning of DBM both within and between countries, to effectively address the unequal impact of globalisation on income and health inequalities demonstrated here and elsewhere.^13^, ^14^

In conclusion, household-level DBM is unequally distributed across groups of household wealth in LMICs, although the direction of this inequality depends on country income. Economic and social globalisation might contribute to DBM, with the impacts of economic globalisation particularly realised by the world's poorest. Double-duty policies that simultaneously address all forms of malnutrition should consider actions that mitigate the potential negative and unequal impacts of globalisation.

## Data sharing

This study used publicly available data that can be found online on their respective repositories: Demographic and Health Survey program (https://dhsprogram.com/), KOF Globalisation Index (https://kof.ethz.ch/en/forecasts-and-indicators/indicators/kof-globalisation-index.html), and the WorldBank (https://data.worldbank.org/). The compiled datasets, analysis files, and logs produced for this study are available from the corresponding author upon request.

**This online publication has been corrected. The corrected version first appeared at thelancet.com/lancetgh on March 15, 2022**

## Declaration of interests

We declare no competing interests.

## Supplementary Materials

Spanish translation of the abstract

Portuguese translation of the abstract

## References

[1] UN (2019). Decade of action on nutrition 2016–2025. https://www.un.org/nutrition/about.

[2] WHO (2017). The double burden of malnutrition: policy brief. https://www.who.int/publications/i/item/WHO-NMH-NHD-17.3.

[3] Popkin BM, Corvalan C, Grummer-Strawn LM (2020). Dynamics of the double burden of malnutrition and the changing nutrition reality. Lancet.

[4] Min J, Zhao Y, Slivka L, Wang Y (2018). Double burden of diseases worldwide: coexistence of undernutrition and overnutrition-related non-communicable chronic diseases. Obes Rev.

[5] Perez-Escamilla R, Bermudez O, Buccini GS (2018). Nutrition disparities and the global burden of malnutrition. BMJ.

[6] Lee J, Houser RF, Must A, De Fulladolsa PP, Bermudez OI (2012). Socioeconomic disparities and the familial coexistence of child stunting and maternal overweight in Guatemala. Econ Hum Biol.

[7] Leroy JL, Habicht JP, de Cossío TG, Ruel MT (2014). Maternal education mitigates the negative effects of higher income on the double burden of child stunting and maternal overweight in rural Mexico. J Nutr.

[8] Parra DC, Gomez LF, Iannotti L, Haire-Joshu D, Sebert Kuhlmann AK, Brownson RC (2018). Multilevel correlates of household anthropometric typologies in Colombian mothers and their infants. Glob Heal Epidemiol Genomics.

[9] Fooken J, Vo LK (2021). Exploring the macroeconomic and socioeconomic determinants of simultaneous over and undernutrition in Asia: an analysis of stunted child - overweight mother households. Soc Sci Med.

[10] Anik AI, Mosfequr Rahman M, Mostafizur Rahman M, Ismail Tareque M, Nuruzzaman Khan M, Mahmudul Alam M (2019). Double burden of malnutrition at household level: a comparative study among Bangladesh, Nepal, Pakistan, and Myanmar. PLoS One.

[11] Oddo VM, Rah JH, Semba RD (2012). Predictors of maternal and child double burden of malnutrition in rural Indonesia and Bangladesh. Am J Clin Nutr.

[12] Gygli S, Haelg F, Potrafke N, Sturm J-E (2019). The KOF Globalisation Index – revisited. Rev Int Organ.

[13] Heimberger P (2020). Does economic globalisation affect income inequality? A meta-analysis. World Econ.

[14] Schrecker T, Labonté R, De Vogli R (2008). Globalisation and health: the need for a global vision. Lancet.

[15] Cuevas García-Dorado S, Cornselsen L, Smith R, Walls H (2019). Economic globalization, nutrition and health: a review of quantitative evidence. Global Health.

[16] Nandi A, Sweet E, Kawachi I, Heymann J, Galea S (2014). Associations between macrolevel economic factors and weight distributions in low- and middle-income countries: a multilevel analysis of 200 000 adults in 40 countries. Am J Public Health.

[17] Neuman M, Kawachi I, Gortmaker S, Subramanian S (2014). National economic development and disparities in body mass index: a cross-sectional study of data from 38 countries. PLoS One.

[18] Barlow P, Loopstra R, Tarasuk V, Reeves A (2020). Liberal trade policy and food insecurity across the income distribution: an observational analysis in 132 countries, 2014–17. Lancet Glob Health.

[19] Watson OJ, FitzJohn R, Eaton JW (2019). rdhs: an R package to interact with The Demographic and Health Surveys (DHS) Program datasets. Wellcome Open Res.

[20] Dieffenbach S, Stein AD (2012). Stunted child/overweight mother pairs represent a statistical artifact, not a distinct entity. J Nutr.

[21] Oberlander L, Disdier A-C, Etilé F (2017). Globalisation and national trends in nutrition and health: a grouped fixed-effects approach to intercountry heterogeneity. Health Econ.

[22] Costa-Font J, Mas N (2016). ‘Globesity’? The effects of globalization on obesity and caloric intake. Food Policy.

[23] Goryakin Y, Lobstein T, James WPT, Suhrcke M (2015). The impact of economic, political and social globalization on overweight and obesity in the 56 low and middle income countries. Soc Sci Med.

[24] De Vogli R, Kouvonen A, Elovainio M, Marmot M (2014). Economic globalization, inequality and body mass index: a cross-national analysis of 127 countries. Crit Public Health.

[25] Hawkes C, Ruel MT, Salm L, Sinclair B, Branca F (2020). Double-duty actions: seizing programme and policy opportunities to address malnutrition in all its forms. Lancet.

[26] Walls H, Smith R, Cuevas S, Hanefeld J (2019). International trade and investment: still the foundation for tackling nutrition related non-communicable diseases in the era of Trump?. BMJ.

[27] Moodie R, Stuckler D, Monteiro C (2013). Profits and pandemics: prevention of harmful effects of tobacco, alcohol, and ultra-processed food and drink industries. Lancet.

[28] Fatima ST, Khan AQ (2019). Globalization and female labor force participation: the role of trading partners. J Int Trade Econ Dev.

[29] Sangha H, Riegler R (2020). Can globalisation promote female labour force participation?. Int J Dev Issues.

[30] Rollins NC, Bhandari N, Hajeebhoy N (2016). Why invest, and what it will take to improve breastfeeding practices?. Lancet.

