# Correction to *Lancet Glob Health* 2022; 10: e482–90

**DOI:** 10.1016/S2214-109X(22)00089-4

**Published:** 2022-03-15

*Seferidi P, Hone T, Duran AC, Bernabe-Ortiz A, Millett C. Global inequalities in the double burden of malnutrition and associations with globalisation: a multilevel analysis of Demographic and Healthy Surveys from 55 low-income and middle-income countries, 1992–2018.* Lancet Glob Health *2022; **10:** e482–90*—In this Article, in the title and abstract, where it reads “Demographic and Healthy Surveys”, it should read “Demographic and Health Surveys”. This correction has been made as of March 15, 2022.

