# Supplementary material for: Global inequalities in the double burden of malnutrition and associations with globalisation: a multilevel analysis of Demographic and Health Surveys from 55 low-income and middle-income countries, 1992–2018
Source: Lancet Glob Health. 2022 Feb 8;10(4):e482–90. doi: 10.1016/S2214-109X(21)00594-5 (PMC8924053; doi:10.1016/S2214-109X(21)00594-5)
Supplement: Spanish translation of the abstract [file mmc1.pdf]

THE LANCET
Global Health

Supplementary appendix 1

This translation in Spanish was submitted by the authors and we reproduce it as supplied. It has not been peer reviewed. *The Lancet*'s editorial processes have only been applied to the original in English, which should serve as reference for this manuscript.

Los autores nos proporcionaron esta traducción al español y la reproducimos tal como nos fue entregada. No la hemos revisado. Los procesos editoriales de *The Lancet* se han aplicado únicamente al original en inglés, que debe servir de referencia para este manuscrito.

Supplement to: Seferidi P, Hone T, Duran AC, Bernabe-Ortiz A, Millett C. Global inequalities in the double burden of malnutrition and associations with globalisation: a multilevel analysis of Demographic and Healthy Surveys from 55 low-income and middle-income countries, 1992–2018. *Lancet Glob Health* 2022; published online Feb 8. https://doi.org/10.1016/S2214-109X(21)00594-5.

Título: Desigualdades globales en la doble carga de malnutrición y asociaciones con la globalización: un análisis multinivel de las Encuestas Demográficas y de Salud de 55 países de bajos y medianos ingresos, 1992–2018

Resumen

Antecedentes

Los países de bajos y medianos ingresos (PBMIs) enfrentan una doble carga de malnutrición (DCM), donde el exceso de peso y la desnutrición coexisten dentro de la misma persona, hogar, o población. Este análisis investiga las desigualdades globales en la DCM a nivel de hogar, definida como la presencia de un niño con retraso en el crecimiento y una madre con sobrepeso, y su asociación con la globalización económica, social y política, según los ingresos del país y la riqueza del hogar.

Métodos

Se combinó los datos antropométricos y demográficos de 1 132 069 niños (<5 años) y sus respectivas madres (15–49 años) de 189 Encuestas Demográficas y de Salud en 55 países de PBMIs entre 1992 y 2018. Estos fueron combinados con datos a nivel de país sobre globalización económica, social, y política según el índice de KOF y el ingreso nacional bruto (INB) según el Banco Mundial. Las asociaciones multivariables entre DCM y la riqueza del hogar, el INB, y la globalización, así como sus interacciones se evaluaron usando modelos de regresión logística multinivel con efectos fijos de país y año y errores estándar robustos agrupados por país.

Hallazgos

La probabilidad de DCM fue mayor en los hogares más ricos en los países más pobres y en los hogares más pobres en los países más ricos de los PBMIs. La globalización económica se asoció con mayor probabilidad de DCM en los hogares más pobres (OR 1·49; IC 95% 1·20–1·86) comparados con los más ricos. Estas asociaciones se atenuaron a medida que aumentaba el INB. La globalización social se asoció a mayor probabilidad de DCM (OR 1·39; IC 95%: 1·16–1·65), independiente de la riqueza del hogar o del ingreso del país. No se identificó asociación entre la globalización política y DCM.

Interpretación

El aumento de la globalización económica y social se asoció a mayores niveles de DCM, aunque el impacto de la globalización económica fue más marcado en los países más pobres del mundo. El patrón económico de DCM observado en este estudio requiere acciones de doble función en poblaciones específicas, que deberían tener como objetivo mitigar los posibles impactos negativos y desiguales de la globalización.
