# Supplementary material for: Global inequalities in the double burden of malnutrition and associations with globalisation: a multilevel analysis of Demographic and Health Surveys from 55 low-income and middle-income countries, 1992–2018
Source: Lancet Glob Health. 2022 Feb 8;10(4):e482–90. doi: 10.1016/S2214-109X(21)00594-5 (PMC8924053; doi:10.1016/S2214-109X(21)00594-5)
Supplement: Portuguese translation of the abstract [file mmc2.pdf]

THE LANCET
Global Health

## Supplementary appendix 2

This translation in Portuguese was submitted by the authors and we reproduce it as supplied. It has not been peer reviewed. *The Lancet*'s editorial processes have only been applied to the original in English, which should serve as reference for this manuscript.

Esta tradução em português foi submetida pelos autores e nós não fizemos quaisquer alterações. Esta versão não foi revista por pares. O processo editorial do The Lancet só foi aplicado à versão original em inglês, que deve servir como referência para este artigo.

Supplement to: Seferidi P, Hone T, Duran AC, Bernabe-Ortiz A, Millett C. Global inequalities in the double burden of malnutrition and associations with globalisation: a multilevel analysis of Demographic and Healthy Surveys from 55 low-income and middle-income countries, 1992–2018. *Lancet Glob Health* 2022; published online Feb 8. https://doi.org/10.1016/S2214-109X(21)00594-5.

**Título:** Desigualdades globais na dupla carga de malnutrição e associações com a globalização: uma análise multinível das Pesquisas Demográficas e de Saúde de 55 países de baixa e média renda, 1992–2018


## Resumo

### Introdução

Os países de renda baixa e média (PRBMs) enfrentam uma dupla carga de malnutrição (DCM), na qual o excesso e o baixo peso coexistem na mesma pessoa, família, ou população. Este estudo investigou as desigualdades globais em DCM em nível domiciliar, definidas como a presença de uma criança de baixa estatura para a idade e uma mãe com excesso de peso, e sua associação com a globalização econômica, social, e política, de acordo com a renda do país e a renda domiciliar.

### Métodos

Combinamos dados demográficos e antropométricos de 1 132 069 crianças (idade <5 anos) e suas respectivas mães (idade 15–49 anos) presentes em 189 Pesquisas Demográficas e de Saúde em 55 PRBMs entre 1992 e 2018. Estes dados foram agregados a dados nacionais de globalização econômica, social, e política do índice Konjunkturforschungsstelle (KOF) de globalização e a renda nacional bruta (RNB) disponibilizada pelo Banco Mundial. Associações multivariadas entre DCM e renda domiciliar, RNB, e globalização, bem como suas interações, foram avaliadas usando modelos multinível de regressão logística com efeitos fixos para o país e ano. Estimamos os erros padrão robustos agrupados por país.

### Resultados

A probabilidade de CMD foi maior nas famílias mais ricas dos países mais pobres e nas famílias mais pobres dos países mais ricos dentre os PBMRs. A globalização econômica foi associada a uma maior probabilidade de DCM nas famílias mais pobres (OR 1·49; IC 95% 1·20–1·86) em comparação com as mais ricas. Essas associações foram atenuadas à medida que o RNB aumentou. A globalização social foi associada a uma maior probabilidade de DCM (OR 1·39; IC 95% 1·16–1·65), independentemente da riqueza familiar ou da renda do país. Não foi identificada associação entre globalização política e DCM.

### Conclusão

O aumento da globalização econômica e social foi associado a níveis mais altos de DCM, embora o impacto da globalização econômica tenha sido mais pronunciado nos países mais pobres do mundo. O padrão econômico do DCM observado neste estudo exige ações de função dupla e focalizadas com o objetivo de mitigar potenciais impactos negativos e desiguais da globalização.
