# Supplementary material for: Global inequalities in the double burden of malnutrition and associations with globalisation: a multilevel analysis of Demographic and Health Surveys from 55 low-income and middle-income countries, 1992–2018
Source: Lancet Glob Health. 2022 Feb 8;10(4):e482–90. doi: 10.1016/S2214-109X(21)00594-5 (PMC8924053; doi:10.1016/S2214-109X(21)00594-5)
Supplement: Supplementary appendix 3 [file mmc3.pdf]

THE LANCET
Global Health

Supplementary appendix 3

This appendix formed part of the original submission and has been peer reviewed. We post it as supplied by the authors.

Supplement to: Seferidi P, Hone T, Duran AC, Bernabe-Ortiz A, Millett C. Global inequalities in the double burden of malnutrition and associations with globalisation: a multilevel analysis of Demographic and Healthy Surveys from 55 low-income and middle-income countries, 1992–2018. *Lancet Glob Health* 2022; published online Feb 8. https://doi.org/10.1016/S2214-109X(21)00594-5.

Supplementary appendix

1. De-normalisation of survey weights

We performed a de-normalisation of the individual DHS survey weights used in our analysis. DHS includes a survey-specific normalisation to its weights, so that the sum of survey weights equals to the survey sample size. Although this is appropriate for single survey analyses, pooled analyses require for survey weights to reflect the real country population. Thus, the DHS program recommends reversing the normalisation process by multiplying the individual survey weights with the population of the country at the time of the survey, divided by the total number of women interviewed for that survey[1]. We used information on the total number of women aged 15-49 in every country and year of our sample from the World Bank[2] to de-normalise the individual survey weights for our analyses.

2. Statistical Analyses

To describe inequalities in the prevalence of DBM across household wealth and country income, we employed a multilevel logistic regression model with an interaction term between wealth index quintiles and GNI (per US$100). The model was adjusted for sociodemographic characteristics described in the manuscript (i.e. breastfeeding mother, urban/rural region, number of children in the household, mother's marital status, child's sex, age of mother (in years), child (in months), and country-level urbanisation and female unemployment). Country and year fixed-effects were used to adjust for time-invariant differences between countries and time-variant global trends shared by all countries. This analytical approach is frequently used when pooling datasets from multiple countries and years and provide robust within-country associations.[3] Robust standard errors clustered by country were used to account for heteroskedasticity of observations across countries and correlation of observations within countries. Using post-regression modelling commands in Stata 15.1, we estimated the average marginal effect for the richest quintile, i.e., the difference in the probability of DBM for child-mother pairs in the richest quintile compared with the poorest quintile, at increasing levels of GNI.

Associations between DBM and globalisation were tested using multilevel logistic regression with country and year fixed-effects and robust standard errors clustered by country. Associations were estimated per 10 units of KOF index change, which approximately indicates the average country change in overall KOF within our sample, to provide more meaningful interpretations. We tested and included interactions with wealth index quintiles and GNI. The final model specification is shown below:

$$\begin{aligned} &\mathrm{Log}[\Pr(DBM_{ijt} = 1)/1 - \Pr(DBM_{ijt} = 1)] \\ &= \beta_0 + \beta_1 KOF_{jt} + \beta_2 GNI_{jt} + \beta_3 WI_{ijt} + \beta_4 KOF_{jt} \times GNI_{jt} + \beta_5 KOF_{jt} \times WI_{ijt} \\ &+ \beta_6 GNI_{jt} \times WI_{ijt} + \beta_7 Covariates_{ijt} + j + t + \varepsilon_{ijt} \end{aligned}$$

where KOF is each globalisation index (per 10 units), GNI is the per capita GNI (per US$100), WI is a dummy variable for wealth index quintiles (taking values 1-5), i is the child-mother pair, j is the country, t is the year, and ε is the error term for unexplained variation.

Adjusted odds ratios (ORs) and 95% confidence intervals (CI) were estimated. Using post-regression modelling, average marginal effects of KOF for each wealth index quintile were estimated at increasing levels of GNI. These were plotted to illustrate associations between KOF and DBM across household wealth and country income. Contrary to performing stratified analysis for low-income and middle-income countries, this approach allows for consideration of within-country income change over time and provides statistical comparisons, while still showing how associations between globalisation and DBM change based on country income levels.

3. Sensitivity analyses

We performed the following sensitivity analyses and robustness checks. First, we tested different time specifications. We tested for non-linear time trends by including a quadratic continuous time variable in the model. We introduced linear time trends for countries with common characteristics, i.e. by region, quartiles of level of urbanisation, and quartiles of female employment, similarly to previous analysis by Oberlander et al.[4] In the most flexible approach, we also tested for linear time trends for each country in the sample to account for country-specific time trends. Second, we run multi-level analyses with random intercepts at country level and with random slopes at region level to account for potential heterogeneity across countries and regions. Moreover, we clustered standard errors for country and wealth index quintiles, using the vcemway command in Stata, to account for potential correlation of observations not only within country but also within wealth index quintiles for each country. We should note that this approach does not consider that wealth index is a relative measure of wealth for each country-year and thus it is not comparable across time. To further test this, we repeated analyses by clustering standard errors for different measures of socioeconomic status, namely living in a rural/urban region and mother's level of education. We also tested potential clustering of standard errors within countries of the same region, performing multiway clustering for both country and region. We tested for non-linear effects for globalisation and GNI by adding quadratic terms of these variables in the model. We performed stratified analysis by sex and age of children (above and below 2 years). We repeated our analysis using normalised survey weights, i.e. weights with constant sum across country-years, which ignore country size and provide the same weight to all countries globally. We fitted models using mother as our level of observation to test the assumption that all observations in each cluster are independent, given that 24.4% of children in the sample had the same mother. We did this, first by defining DBM as an overweight mother with at least one stunted child and second by randomly selecting one child per mother to include in our sample. We performed sensitivity analysis using 23kg/m^2 as the overweight cut-off point for mothers in South Asian countries in our sample (Bangladesh, India, Maldives, Nepal, Pakistan), as this has been suggested to better reflect true associations with adiposity and health in that population[5]. As the composition of wealth index quintiles is likely to be impacted by the level of globalisation in the country, we also tested interactions with other pre-determined socioeconomic characteristics, namely urban/rural area and mother's education. Finally, we included as model covariates the prevalence of child stunting and mother overweight estimated by country, year, urban/rural region, and wealth index quintile group. We also performed dominance analysis to estimate the relative importance of these variables in the model fit by comparing the Pseudo R^2 model-fit statistics across multiple models, using the domin command in Stata. This is to address concerns that associations with stunted child-overweight mother pairs are a statistical artifact attributed to the underlying trends of overweight and stunting in the population and are not a distinct issue.[6] Adjusting for these covariates provides evidence on the robustness of associations with DBM regardless of the underlying prevalence of stunting and overweight, while the dominance analysis quantifies the relative contribution of these underlying trends in the observed associations.[7]

Supplementary appendix references

1. Ren, R. Note on DHS standard weight de-normalization. *DHS Program User Forum* https://userforum.dhsprogram.com/index.php?t=getfile&id=4& (2013).

2. DataBank. World Development Indicators. Population ages 15-64, female (% of female population). https://databank.worldbank.org/reports.aspx?source=2&series=SP.POP.1564.FE.ZS&country=#.

3. Templin, T., Cravo Oliveira Hashiguchi, T., Thomson, B., Dieleman, J. & Bendavid, E. The overweight and obesity transition from the wealthy to the poor in low- and middle-income countries: A survey of household data from 103 countries. *PLOS Med.* **16**, e1002968 (2019).

4. Oberlander, L., Disdier, A. C. & Etilé, F. Globalisation and national trends in nutrition and health: A grouped fixed-effects approach to intercountry heterogeneity. *Heal. Econ. (United Kingdom)* **26**, 1146–1161 (2017).

5. Nishida, C. *et al.* Appropriate body-mass index for Asian populations and its implications for policy and intervention strategies. *Lancet* **363**, 157–163 (2004).

6. Dieffenbach, S. & Stein, A. D. Stunted Child/Overweight Mother Pairs Represent a Statistical Artifact, Not a Distinct Entity. *J. Nutr.* **142**, 771–773 (2012).

7. Fooken, J. & Vo, L. K. Exploring the macroeconomic and socioeconomic determinants of simultaneous over and undernutrition in Asia: An analysis of stunted child - overweight mother households. *Soc. Sci. Med.* **269**, 113570 (2021).

4. Supplementary tables and figures

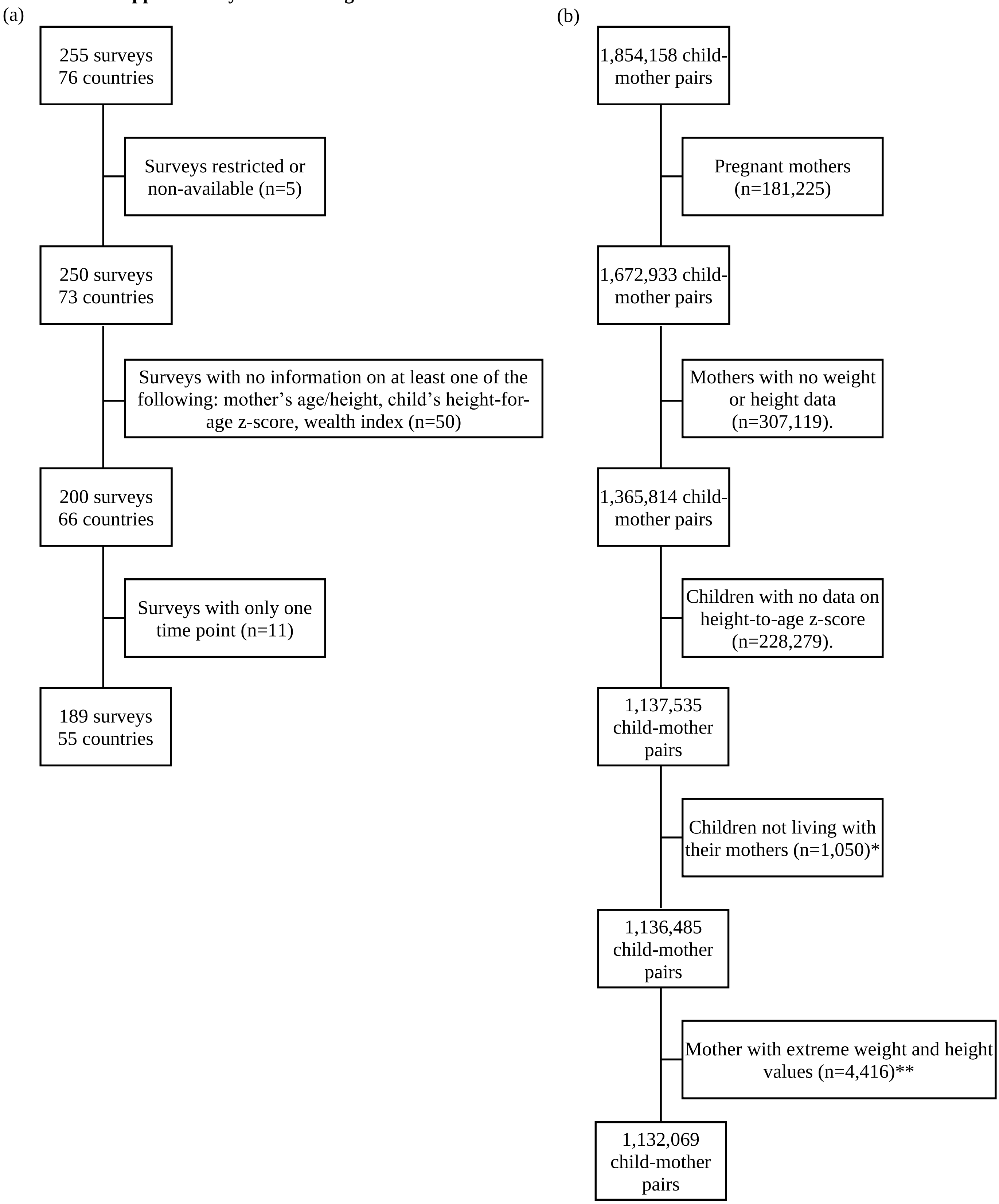

Supplementary Figure 1. Flow charts with (a) survey and (b) child-mother pair exclusions
**Where this information was missing (n=4,459), we assumed that the children were living with their mother*
***Extreme values were those below the 0.01th and above the 99.9th percentile, which were 30.7 kg and 119.8 kg for weight and 117 cm and 181 cm for height, respectively. No outliers were identified for children's height-for-age z-scores.*

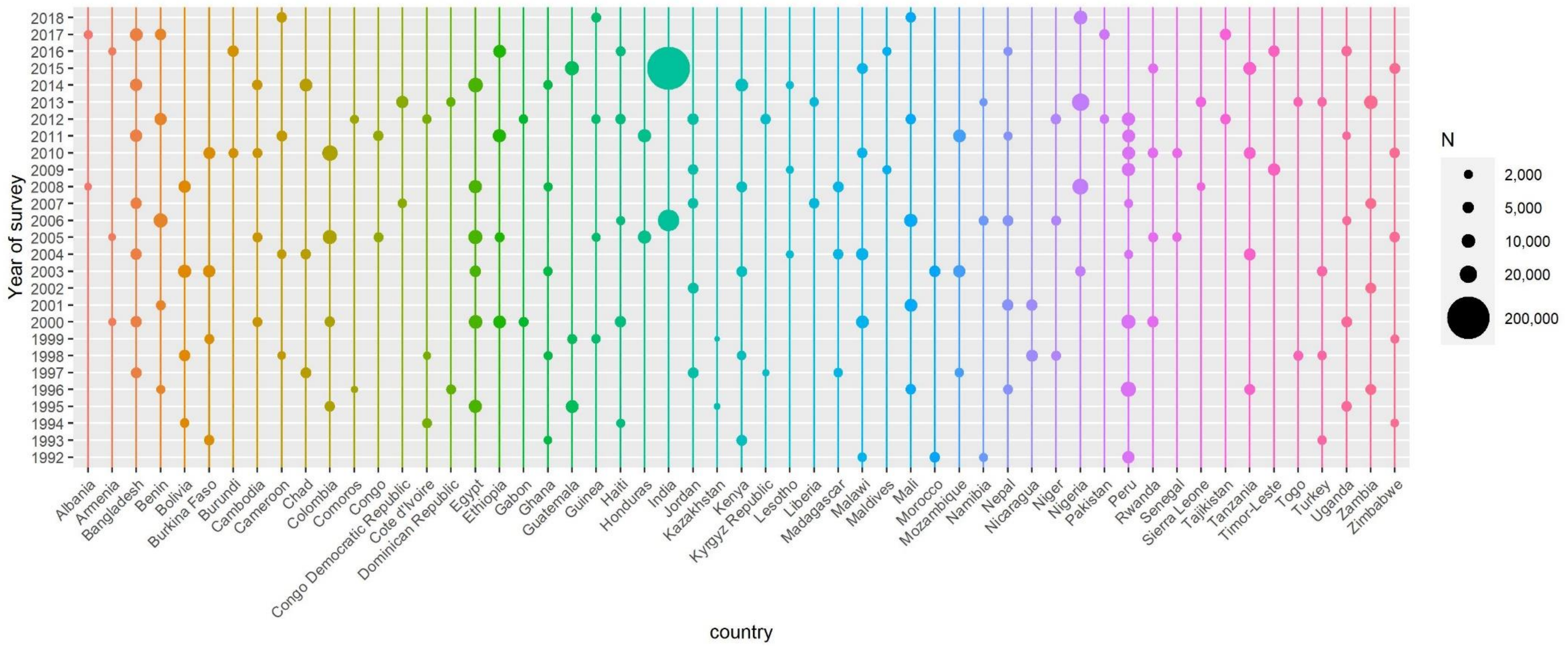

Supplementary Figure 2. Countries and years of surveys included in the sample.
The size of the points represents the sample size of each survey.

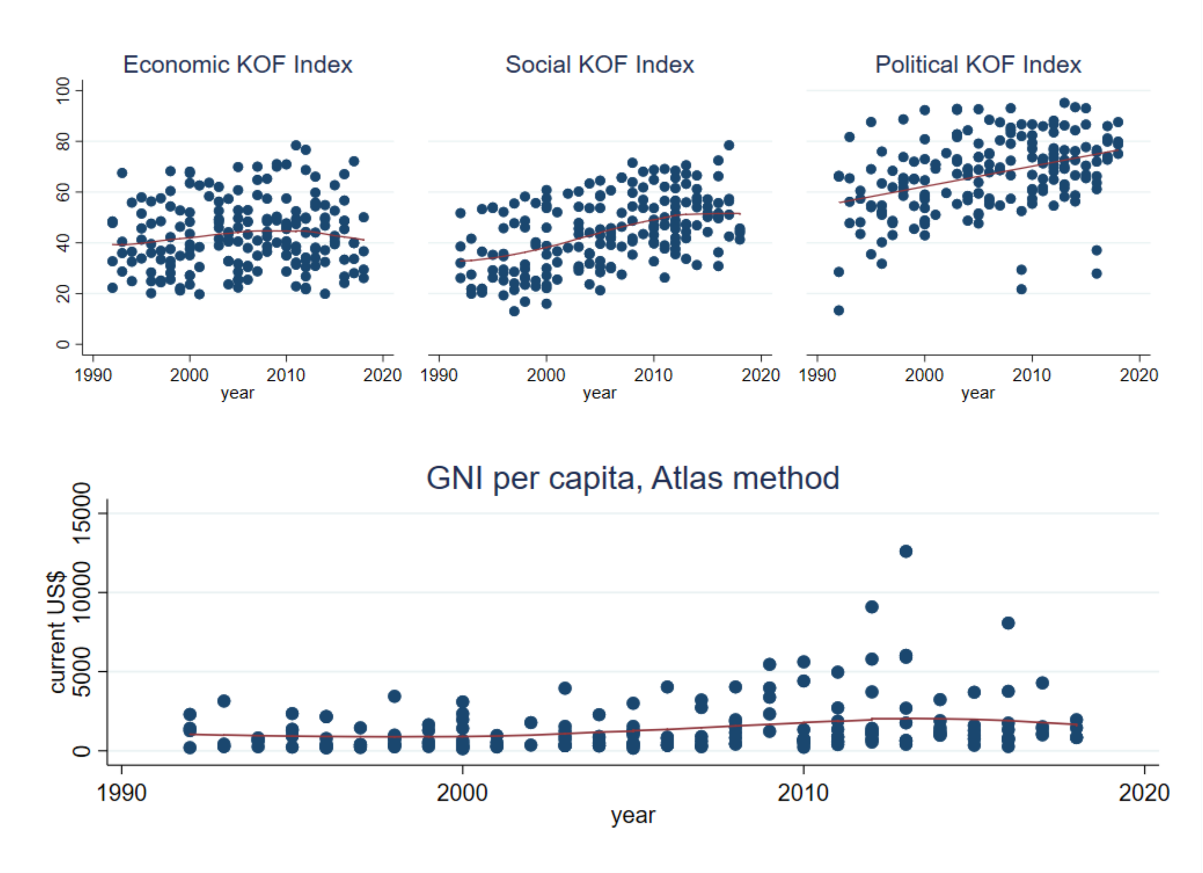

Supplementary Figure 3. Scatterplots with lowess prediction for Economic, Social, and Political KOF Indices and Gross National Income per capita, over time between 1992 and 2018.

Supplementary Table 1. Associations between the Economic KOF Globalisation Index and its two subcomponents (Trade KOF and Financial KOF) and the double burden of malnutrition (stunted child with overweight mother) and their interactions with wealth index quintile and Gross National Income per capita.

	Economic KOF	Trade KOF	Financial KOF
KOF	1.49***	1.24*	1.27***
	[1.20,1.86]	[1.04,1.47]	[1.11,1.45]
Interactions with wealth index quintiles			
Poorest * KOF	1.00	1.00	1.00
	[1.00,1.00]	[1.00,1.00]	[1.00,1.00]
Poorer * KOF	0.96	0.97	0.98
	[0.89,1.04]	[0.89,1.04]	[0.93,1.04]
Middle * KOF	0.84*	0.90	0.90*
	[0.72,0.99]	[0.77,1.04]	[0.80,1.00]
Richer * KOF	0.80**	0.86	0.87*
	[0.68,0.93]	[0.71,1.03]	[0.78,0.97]
Richest * KOF	0.73***	0.82	0.81***
	[0.64,0.83]	[0.67,1.00]	[0.73,0.90]
Interactions with Gross National Income			
GNI * KOF	0.99*	0.99*	1.00*
	[0.99,1.00]	[0.99,1.00]	[0.99,1.00]

Models are adjusted for wealth index quintiles, GNI, wealth index quintiles and GNI interaction, country and year fixed-effects, breastfeeding mother, urban/rural region, number of children in the household, mother's marital status, child's sex, age of mother (in years), child (in months), country-level urbanisation and female unemployment. SEs are clustered by country.
* $p < 0.05$, ** $p < 0.01$, *** $p < 0.001$

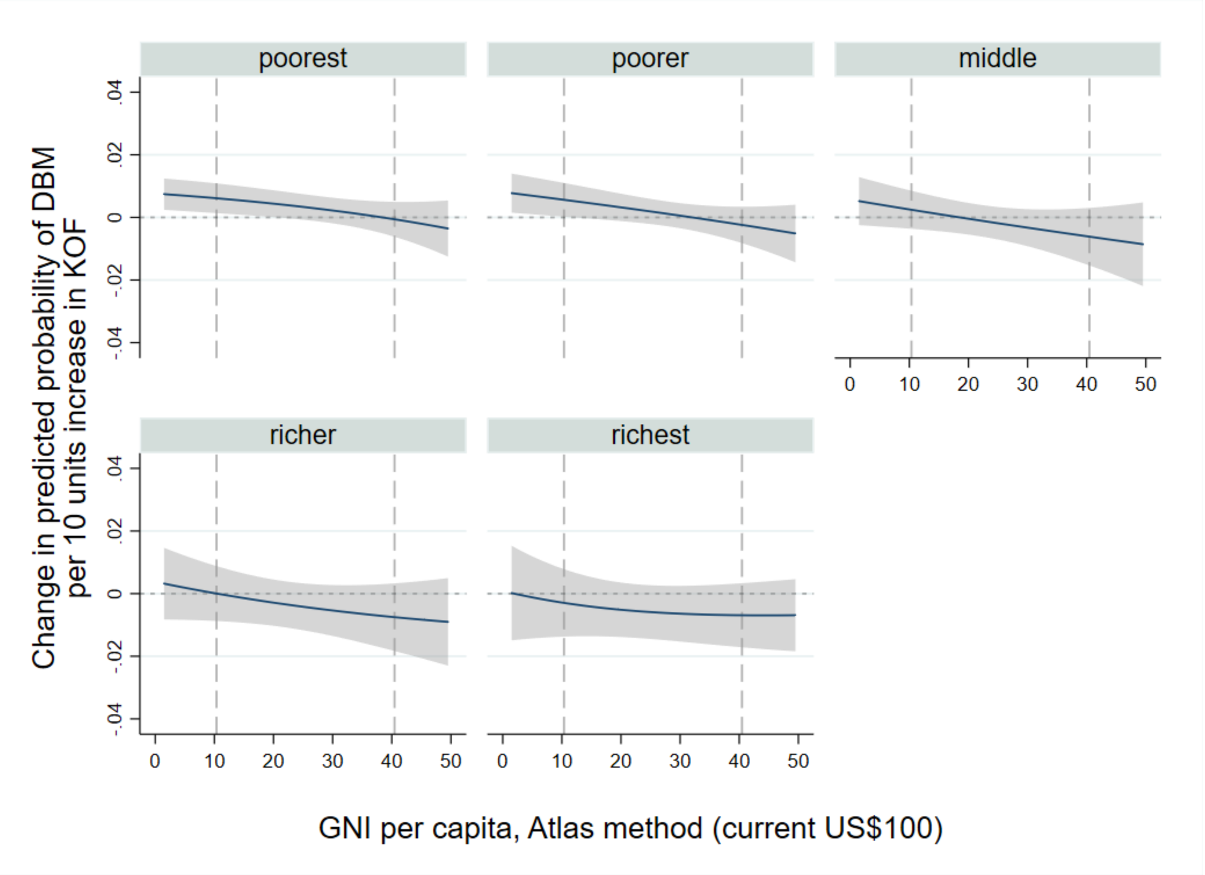

Supplementary Figure 4. Associations between KOF Trade Index and the Double Burden of Malnutrition across wealth index quintiles (poorest, poorer, middle, richer, richest), as Gross National Income (GNI) per capita increases

Average Marginal Effects have been calculated at GNI for every $US100 between US$150 and US$4950
Vertical dashed lines indicate the cut-off points for lower-middle income countries (US$1,036) and upper-middle income countries (US$ 4,046), as defined by the World Bank in 2021.
The shaded area denotes 95% Confidence Intervals.

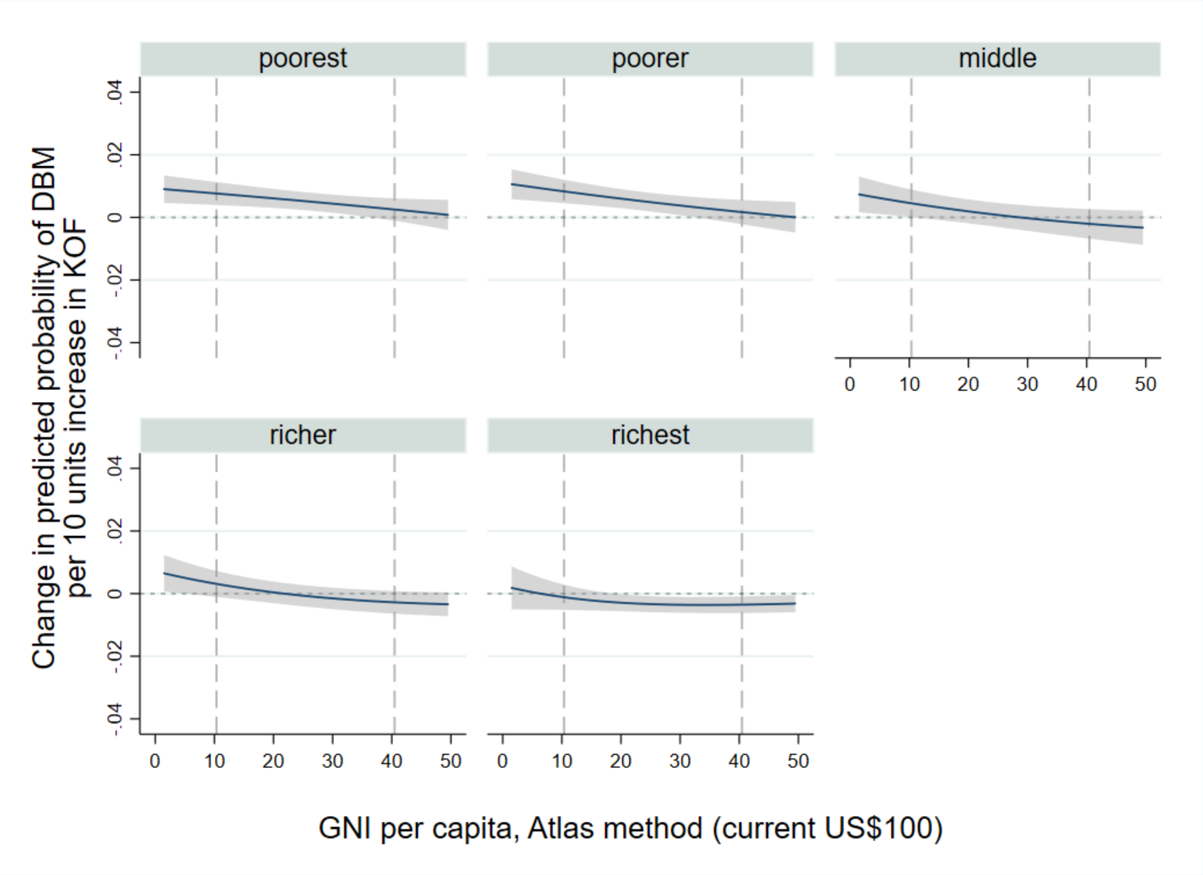

Supplementary Figure 5. Associations between KOF Financial Index and the Double Burden of Malnutrition across wealth index quintiles (poorest, poorer, middle, richer, richest), as Gross National Income (GNI) per capita increases

Average Marginal Effects have been calculated at GNI for every $US100 between US$150 and US$4950

Vertical dashed lines indicate the cut-off points for lower-middle income countries (US$1,036) and upper-middle income countries (US$ 4,046), as defined by the World Bank in 2021.

The shaded area denotes 95% Confidence Intervals.

Supplementary Table 2. Associations between the Social KOF Globalisation Index and its three subcomponents (Interpersonal KOF, Informational KOF, Cultural KOF) (per 10 units increase) and the double burden of malnutrition (stunted child with overweight mother) and their interactions with wealth index quintile and Gross National Income per capita.

	**Social KOF**	**Interpersonal KOF**	**Informational KOF**	**Cultural KOF**
KOF	1.39***	1.41***	1.24*	0.99
	[1.16,1.65]	[1.16,1.71]	[1.02,1.52]	[0.81,1.22]
Interactions with wealth index quintiles				
Poorest * KOF	1.00	1.00	1.00	1.00
	[1.00,1.00]	[1.00,1.00]	[1.00,1.00]	[1.00,1.00]
Poorer * KOF	1.06	0.98	1.10*	1.04
	[0.99,1.13]	[0.90,1.06]	[1.00,1.20]	[0.96,1.13]
Middle * KOF	1.06	0.90	1.12	1.10
	[0.91,1.23]	[0.77,1.05]	[0.95,1.32]	[0.91,1.32]
Richer * KOF	1.10	0.87	1.15	1.15
	[0.93,1.29]	[0.71,1.07]	[0.94,1.41]	[0.94,1.40]
Richest * KOF	1.10	0.81	1.18	1.20
	[0.91,1.33]	[0.65,1.02]	[0.92,1.50]	[0.97,1.48]
Interactions with Gross National Income				
GNI * KOF	1.00	1.00	1.00	1.00
	[0.99,1.01]	[0.99,1.01]	[0.99,1.00]	[1.00,1.01]

Models are adjusted for wealth index quintiles, GNI, wealth index quintiles and GNI interaction, country and year fixed-effects, breastfeeding mother, urban/rural region, number of children in the household, mother's marital status, child's sex, age of mother (in years),child (in months), country-level urbanisation and female unemployment. SEs are clustered by country.
* $p < 0.05$, ** $p < 0.01$, *** $p < 0.001$

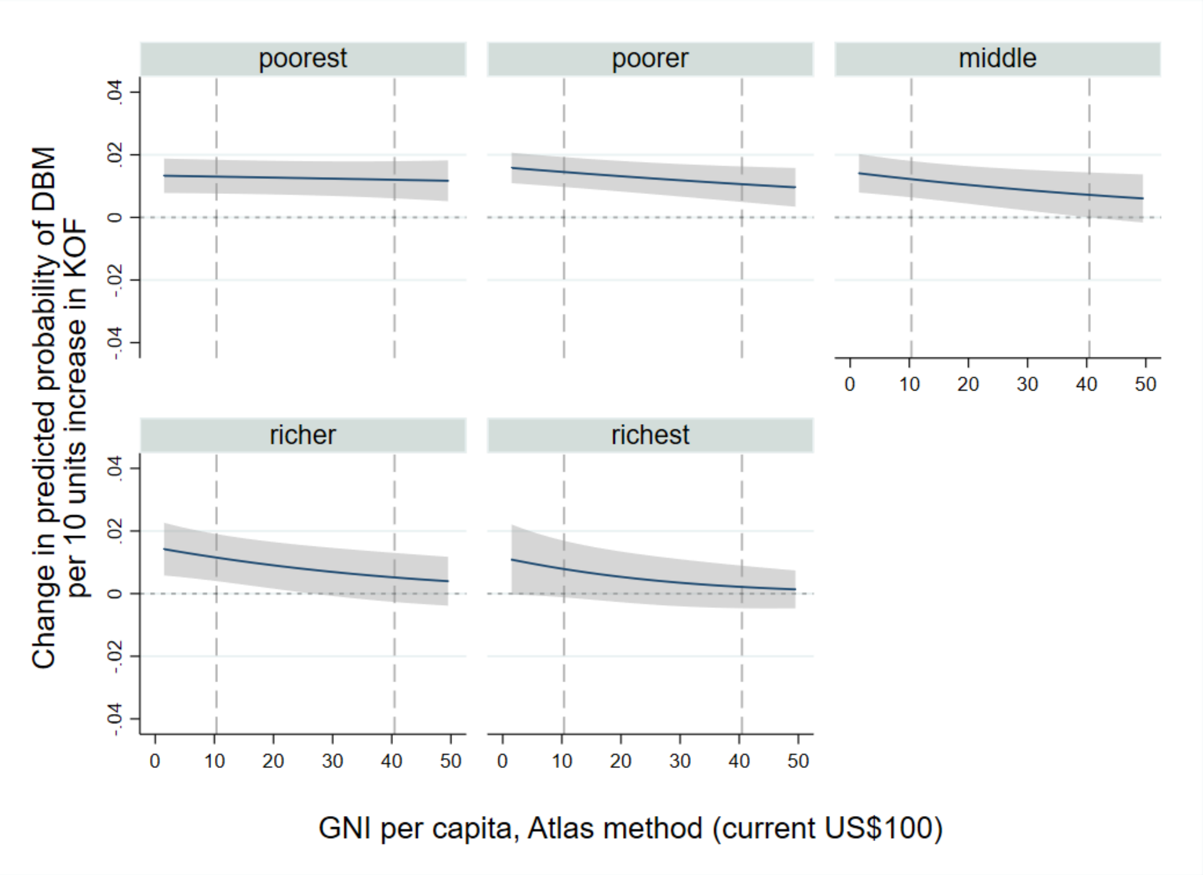

Supplementary Figure 6. Associations between KOF Interpersonal Index and the Double Burden of Malnutrition across wealth index quintiles (poorest, poorer, middle, richer, richest), as Gross National Income (GNI) per capita increases

Average Marginal Effects have been calculated at GNI for every $US100 between US$150 and US$4950
Vertical dashed lines indicate the cut-off points for lower-middle income countries (US$1,036) and upper-middle income countries (US$ 4,046), as defined by the World Bank in 2021.
The shaded area denotes 95% Confidence Intervals.

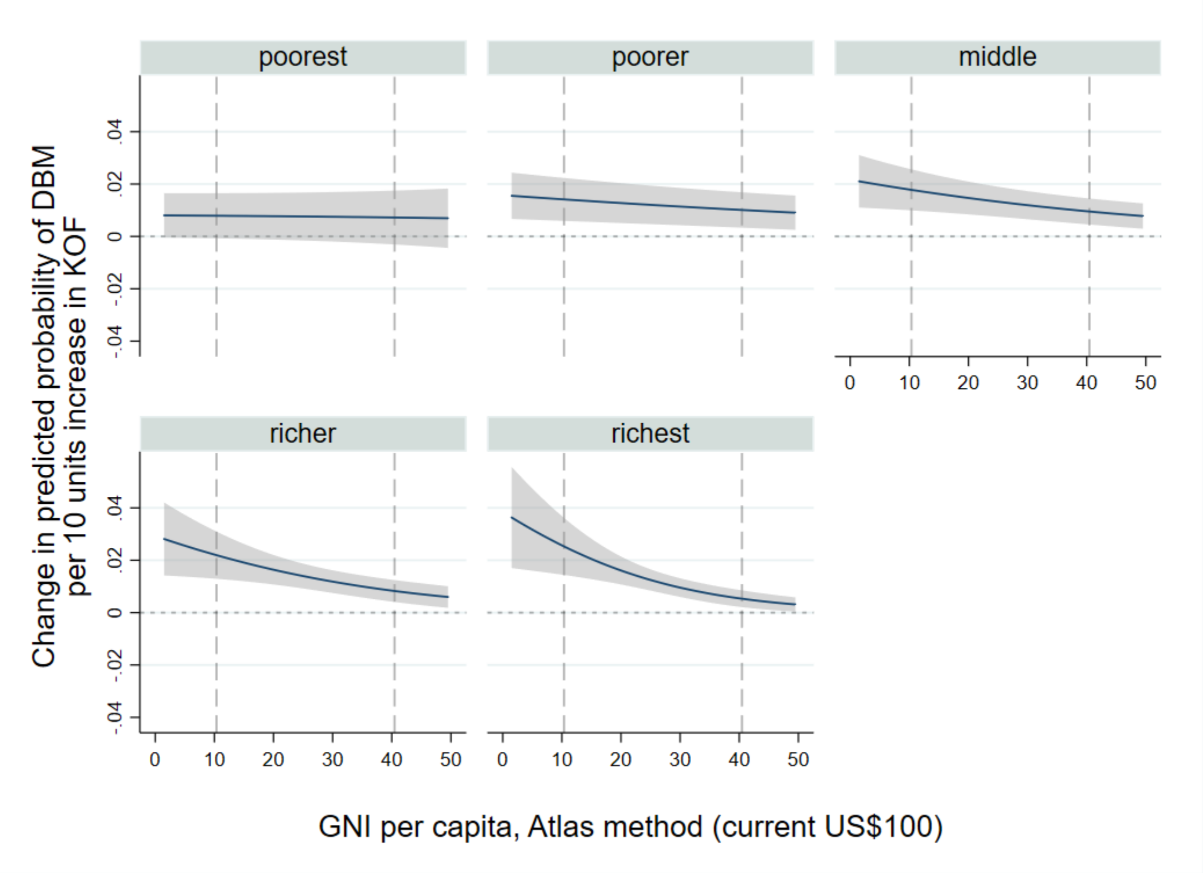

Supplementary Figure 7. Associations between KOF Informational Index and the Double Burden of Malnutrition across wealth index quintiles (poorest, poorer, middle, richer, richest), as Gross National Income (GNI) per capita increases

Average Marginal Effects have been calculated at GNI for every $US100 between US$150 and US$4950
Vertical dashed lines indicate the cut-off points for lower-middle income countries (US$1,036) and upper-middle income countries (US$ 4,046), as defined by the World Bank in 2021.
The shaded area denotes 95% Confidence Intervals.

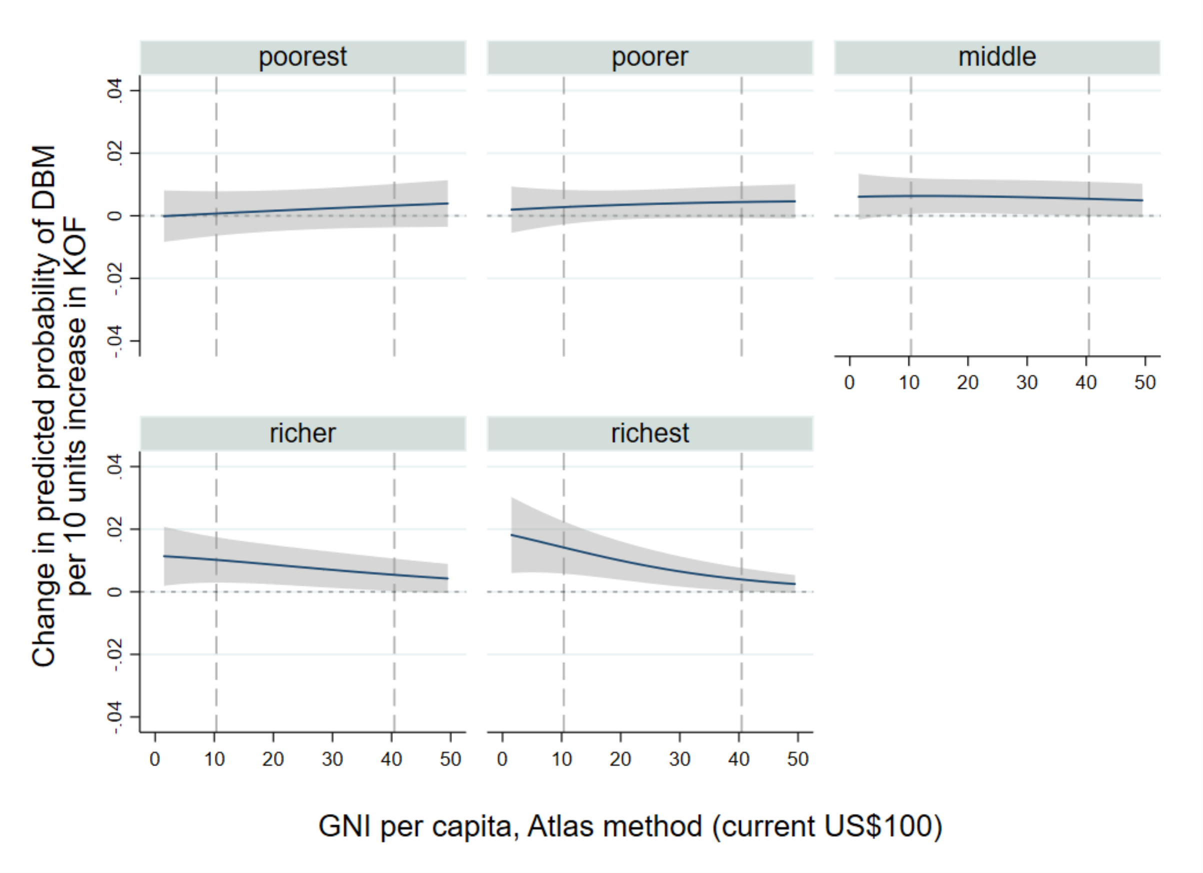

Supplementary Figure 8. Associations between KOF Cultural Index and the Double Burden of Malnutrition across wealth index quintiles (poorest, poorer, middle, richer, richest), as Gross National Income (GNI) per capita increases

Average Marginal Effects have been calculated at GNI for every $US100 between US$150 and US$4950
Vertical dashed lines indicate the cut-off points for lower-middle income countries (US$1,036) and upper-middle income countries (US$ 4,046), as defined by the World Bank in 2021.
The shaded area denotes 95% Confidence Intervals.

Supplementary Table 3. Associations between the KOF Globalisation Index (per 10 units increase) and the double burden of malnutrition (stunted child with overweight mother) and their interactions with wealth index quintile and Gross National Income per capita under different time specifications

	Quadratic time trend	Time trends by region	Time trends by urbanisation quartiles	Time trends by female unemployment quartiles	Time trends by country
Economic KOF					
KOF	1.34**	1.33***	1.48***	1.51***	1.28**
	[1.08,1.67]	[1.14,1.56]	[1.21,1.81]	[1.22,1.87]	[1.10,1.50]
Interactions with wealth index quintiles					
Poorest * KOF	1.00	1.00	1.00	1.00	1.00
	[1.00,1.00]	[1.00,1.00]	[1.00,1.00]	[1.00,1.00]	[1.00,1.00]
Poorer * KOF	0.96	0.96	0.96	0.96	0.96
	[0.89,1.04]	[0.89,1.04]	[0.89,1.04]	[0.89,1.04]	[0.89,1.04]
Middle * KOF	0.83*	0.84*	0.84*	0.84*	0.84*
	[0.70,0.99]	[0.71,0.99]	[0.71,0.99]	[0.71,0.99]	[0.71,0.99]
Richer * KOF	0.78**	0.79**	0.80**	0.79**	0.79**
	[0.67,0.92]	[0.68,0.93]	[0.68,0.93]	[0.68,0.93]	[0.68,0.93]
Richest * KOF	0.71***	0.72***	0.73***	0.72***	0.72***
	[0.62,0.83]	[0.63,0.83]	[0.63,0.83]	[0.63,0.83]	[0.63,0.83]
Interactions with Gross National Income					
GNI * KOF	0.99**	1.00*	0.99**	1.00	1.00
	[0.98,1.00]	[0.99,1.00]	[0.99,1.00]	[0.99,1.01]	[1.00,1.01]
Social KOF					
KOF	1.20	1.12	1.36***	1.38***	0.97
	[0.96,1.51]	[0.96,1.29]	[1.15,1.62]	[1.17,1.62]	[0.78,1.20]
Interactions with wealth index quintiles					
Poorest * KOF	1.00	1.00	1.00	1.00	1.00
	[1.00,1.00]	[1.00,1.00]	[1.00,1.00]	[1.00,1.00]	[1.00,1.00]
Poorer * KOF	1.06	1.06	1.06	1.06	1.05
	[0.99,1.13]	[0.99,1.13]	[0.99,1.13]	[0.99,1.13]	[0.98,1.13]
Middle * KOF	1.05	1.06	1.06	1.05	1.05
	[0.91,1.21]	[0.91,1.23]	[0.91,1.23]	[0.91,1.23]	[0.90,1.23]
Richer * KOF	1.08	1.09	1.10	1.09	1.09
	[0.93,1.26]	[0.92,1.29]	[0.93,1.29]	[0.92,1.29]	[0.92,1.29]
Richest * KOF	1.08	1.10	1.10	1.10	1.09
	[0.90,1.28]	[0.90,1.33]	[0.91,1.34]	[0.90,1.33]	[0.89,1.33]
Interactions with Gross National Income					
GNI * KOF	0.99	1.00	0.99**	1.00	1.01
	[0.98,1.00]	[0.99,1.00]	[0.99,1.00]	[0.99,1.01]	[1.00,1.02]
Political KOF					
KOF	0.71*	1.01	0.85	0.89	1.01
	[0.51,0.99]	[0.79,1.30]	[0.65,1.11]	[0.68,1.17]	[0.79,1.29]
Interactions with wealth index quintiles					
Poorest * KOF	1.00	1.00	1.00	1.00	1.00
	[1.00,1.00]	[1.00,1.00]	[1.00,1.00]	[1.00,1.00]	[1.00,1.00]
Poorer * KOF	1.13**	1.13**	1.13**	1.13**	1.13**
	[1.05,1.22]	[1.05,1.22]	[1.05,1.22]	[1.05,1.22]	[1.05,1.22]
Middle * KOF	1.15	1.16	1.16	1.16	1.16
	[0.95,1.40]	[0.96,1.41]	[0.96,1.41]	[0.95,1.41]	[0.96,1.42]
Richer * KOF	1.16	1.18	1.18	1.17	1.18
	[0.91,1.48]	[0.91,1.51]	[0.92,1.51]	[0.91,1.51]	[0.91,1.52]
Richest * KOF	1.13	1.15	1.15	1.14	1.15
	[0.82,1.56]	[0.82,1.61]	[0.82,1.61]	[0.82,1.60]	[0.81,1.62]
Interactions with Gross National Income					
GNI * KOF	0.99	1.00	1.00	1.00	1.01
	[0.98,1.00]	[0.99,1.00]	[1.00,1.01]	[1.00,1.01]	[0.99,1.02]

* $p < 0.05$, ** $p < 0.01$, *** $p < 0.001$

Supplementary Table 4. Associations between the KOF Globalisation Index (per 10 units increase) and the double burden of malnutrition (stunted child with overweight mother) and their interactions with wealth index quintile and Gross National Income per capita in mixed-effects models

	Economic KOF		*Social KOF*		*Political KOF*	
	Random intercepts	*Random slopes by country*	*Random intercepts*	*Random slopes by country*	*Random intercepts*	*Random slopes by country*
KOF	1.81***	1.89***	1.30***	1.31**	1.19	1.13
	[1.58,2.06]	[1.67,2.14]	[1.13,1.48]	[1.11,1.55]	[0.99,1.43]	[0.96,1.32]
Interactions with wealth index quintiles						
Poorest * KOF	0.96	0.95	1.00	1.00	1.00	1.00
	[0.89,1.03]	[0.89,1.02]	[1.00,1.00]	[1.00,1.00]	[1.00,1.00]	[1.00,1.00]
Poorer * KOF	0.84*	0.83*	1.01	1.05	1.14**	1.22***
	[0.71,0.98]	[0.72,0.97]	[0.94,1.08]	[0.96,1.14]	[1.04,1.25]	[1.11,1.33]
Middle * KOF	0.79**	0.79***	0.99	1.04	1.16	1.21
	[0.68,0.92]	[0.68,0.91]	[0.86,1.15]	[0.88,1.24]	[0.94,1.43]	[0.99,1.47]
Richer * KOF	0.72***	0.72***	1.03	1.06	1.17	1.19
	[0.63,0.82]	[0.63,0.81]	[0.88,1.20]	[0.88,1.28]	[0.90,1.52]	[0.93,1.52]
	0.96	0.95				
Richest * KOF	[0.89,1.03]	[0.89,1.02]	1.05	1.06	1.14	1.14
			[0.89,1.25]	[0.85,1.32]	[0.80,1.61]	[0.82,1.59]
Interactions with Gross National Income						
GNI * KOF	0.99***	0.99***	0.99***	0.99***	0.99***	0.99***
	0.98,0.99	0.98,0.99	[0.98,0.99]	[0.98,0.99]	[0.99,1.00]	[0.99,1.00]

* p < 0.05, ** p < 0.01, *** p < 0.001

Supplementary Table 5. Associations between the KOF Globalisation Index (per 10 units increase) and the double burden of malnutrition (stunted child with overweight mother) and their interactions with wealth index quintile and Gross National Income per capita, performing multiway clustering of standard errors by country and socioeconomic determinants

	Clustered by country and wealth index	*Clustered by country and rural/urban area*	*Clustered by country and mother education*	*Clustered by country and region*
Economic KOF				
KOF	1.49^{***}	1.49^{**}	1.49^{***}	1.49^{*}
	[1.21,1.85]	[1.18,1.89]	[1.23,1.82]	[1.03,2.16]
Interactions with wealth index quintiles				
Poorest * KOF	1.00	1.00	1.00	1.00
	[1.00,1.00]	[1.00,1.00]	[1.00,1.00]	[1.00,1.00]
Poorer * KOF	0.96^{*}	0.96	0.96	0.96
	[0.93,1.00]	[0.87,1.07]	[0.89,1.05]	[0.89,1.05]
Middle * KOF	0.84^{*}	0.84	0.84^{**}	0.84
	[0.73,0.97]	[0.67,1.06]	[0.75,0.94]	[0.69,1.02]
Richer * KOF	0.80^{***}	0.80	0.80^{**}	0.80^{**}
	[0.70,0.91]	[0.60,1.05]	[0.68,0.94]	[0.67,0.94]
Richest * KOF	0.73^{***}	0.73^{***}	0.73^{***}	0.73^{**}
	[0.64,0.83]	[0.61,0.87]	[0.66,0.80]	[0.60,0.89]
Interactions with Gross National Income				
GNI * KOF	0.99^{**}	0.99^{***}	0.99^{***}	0.99
	[0.99,1.00]	[0.99,1.00]	[0.99,1.00]	[0.98,1.00]
Social KOF				
KOF	1.39^{***}	1.39^{***}	1.39^{***}	1.39^{***}
	[1.20,1.60]	[1.20,1.60]	[1.22,1.57]	[1.24,1.55]
Interactions with wealth index quintiles				
Poorest * KOF	1.00	1.00	1.00	1.00
	[1.00,1.00]	[1.00,1.00]	[1.00,1.00]	[1.00,1.00]
Poorer * KOF	1.06^{**}	1.06^{*}	1.06^{*}	1.06
	[1.02,1.10]	[1.01,1.10]	[1.00,1.11]	[0.95,1.17]
Middle * KOF	1.06	1.06	1.06	1.06
	[0.94,1.19]	[0.89,1.26]	[0.90,1.25]	[0.86,1.31]
Richer * KOF	1.10	1.10	1.10	1.10
	[0.94,1.27]	[0.81,1.49]	[0.95,1.26]	[0.88,1.37]
Richest * KOF	1.10	1.10	1.10	1.10
	[0.93,1.31]	[0.91,1.34]	[0.97,1.25]	[0.90,1.35]
Interactions with Gross National Income				
GNI * KOF	1.00	1.00	1.00	1.00
	[0.99,1.01]	[0.99,1.01]	[0.99,1.01]	[1.00,1.00]
Political KOF				
KOF	0.88	0.88	0.88	0.88
	[0.71,1.08]	[0.76,1.02]	[0.70,1.09]	[0.67,1.14]
Interactions with wealth index quintiles				
Poorest * KOF	1.00	1.00	1.00	1.00
	[1.00,1.00]	[1.00,1.00]	[1.00,1.00]	[1.00,1.00]
Poorer * KOF	1.14^{***}	1.14^{**}	1.14^{***}	1.14^{**}
	[1.07,1.20]	[1.05,1.23]	[1.07,1.20]	[1.04,1.24]
Middle * KOF	1.16^{*}	1.16^{*}	1.16^{*}	1.16
	[1.02,1.33]	[1.00,1.35]	[1.02,1.32]	[0.95,1.43]
Richer * KOF	1.18	1.18	1.18	1.18
	[0.97,1.42]	[0.98,1.41]	[0.99,1.39]	[0.90,1.54]
Richest * KOF	1.15	1.15	1.15	1.15
	[0.88,1.49]	[0.90,1.46]	[0.90,1.46]	[0.81,1.63]
Interactions with Gross National Income				
GNI * KOF	1.00	1.00	1.00^{***}	1.00
	[1.00,1.01]	[1.00,1.01]	[1.00,1.00]	[1.00,1.01]

* $p < 0.05$, ** $p < 0.01$, *** $p < 0.001$

Supplementary Table 6. Associations between the KOF Globalisation Index (per 10 units increase) and the double burden of malnutrition (stunted child with overweight mother) and their interactions with wealth index quintile and Gross National Income per capita (GNI). adjusting by quadratic KOF and GNI terms

	Economic KOF			*Social KOF*			*Political KOF*		
	Quadratic GNI	*Quadratic KOF*	*Quadratic GNI and KOF*	*Quadratic GNI*	*Quadratic KOF*	*Quadratic GNI and KOF*	*Quadratic GNI*	*Quadratic KOF*	*Quadratic GNI and KOF*
KOF	1.51***	1.67	1.36	1.40***	1.45	1.22	0.86	1.05	0.99
	[1.22,1.87]	[0.96,2.94]	[0.83,2.23]	[1.17,1.67]	[0.91,2.33]	[0.85,1.76]	[0.69,1.08]	[0.61,1.81]	[0.68,1.45]
KOF*KOF		0.99	1.01		0.99	1.02	0.98	0.99	0.98
		[0.94,1.04]	[0.97,1.06]		[0.94,1.05]	[0.97,1.07]	[0.95,1.02]	[0.96,1.02]	[0.95,1.02]
Gross National Income									
GNI	1.04	1.04	1.04	1.00	1.00	1.01	0.98	0.97	0.97
	[0.99,1.08]	[1.00,1.09]	[1.00,1.09]	[0.94,1.06]	[0.91,1.09]	[0.94,1.09]	[0.93,1.03]	[0.91,1.03]	[0.93,1.02]
*GNI*GNI*	1.00*		1.00**	1.00		1.00	1.00		1.00
	[1.00,1.00]		[1.00,1.00]	[1.00,1.00]		[1.00,1.00]	[1.00,1.00]		[1.00,1.00]
Interactions with wealth index quintiles									
Poorest * KOF	1.00	1.00	1.00	1.06	1.06	1.05	1.00	1.00	1.00
	[1.00,1.00]	[1.00,1.00]	[1.00,1.00]	[0.99,1.13]	[0.99,1.13]	[0.98,1.13]	[1.00,1.00]	[1.00,1.00]	[1.00,1.00]
Poorer * KOF	0.96	0.96	0.96	1.05	1.06	1.05	1.13**	1.14***	1.14**
	[0.89,1.04]	[0.89,1.04]	[0.89,1.04]	[0.90,1.23]	[0.91,1.23]	[0.90,1.23]	[1.05,1.22]	[1.05,1.22]	[1.05,1.23]
Middle * KOF	0.84*	0.84*	0.84*	1.09	1.09	1.09	1.16	1.16	1.16
	[0.71,0.99]	[0.71,0.99]	[0.71,0.99]	[0.92,1.30]	[0.93,1.30]	[0.92,1.29]	[0.95,1.41]	[0.96,1.41]	[0.95,1.42]
Richer * KOF	0.79**	0.80**	0.79**	1.10	1.10	1.10	1.17	1.18	1.17
	[0.67,0.92]	[0.68,0.93]	[0.68,0.92]	[0.90,1.34]	[0.91,1.34]	[0.90,1.34]	[0.91,1.51]	[0.92,1.51]	[0.91,1.52]
				1.00	1.00	1.00			
Richest * KOF	0.72***	0.73***	0.72***	[1.00,1.00]	[1.00,1.00]	[1.00,1.00]	1.14	1.15	1.15
	[0.63,0.83]	[0.63,0.84]	[0.63,0.83]				[0.81,1.61]	[0.82,1.61]	[0.81,1.62]
Interactions with Gross National Income									
GNI * KOF	0.99**	0.99	0.99*	1.00	1.00	1.00	1.00	1.00	1.00
	[0.99,1.00]	[0.99,1.00]	[0.99,1.00]	[0.99,1.01]	[0.99,1.01]	[0.99,1.01]	[1.00,1.01]	[1.00,1.01]	[1.00,1.01]

* $p < 0.05$, ** $p < 0.01$, *** $p < 0.001$

Supplementary Table 7. Associations between the KOF Globalisation Index (per 10 units increase) and the double burden of malnutrition (stunted child with overweight mother) and their interactions with wealth index quintile and Gross National Income per capita, stratified by child's age and sex

	Boys	*Girls*	*Children under 2 years*	*Children over 2 years*
Economic KOF				
KOF	1.58*** [1.27,1.96]	1.44** [1.16,1.79]	1.57*** [1.20,2.04]	1.51*** [1.21,1.88]
Interactions with wealth index quintiles				
Poorest * KOF	1.00 [1.00,1.00]	1.00 [1.00,1.00]	1.00 [1.00,1.00]	1.00 [1.00,1.00]
Poorer * KOF	0.97 [0.89,1.06]	0.95 [0.86,1.05]	0.99 [0.90,1.10]	0.95 [0.88,1.02]
Middle * KOF	0.83* [0.69,1.00]	0.85* [0.73,0.98]	0.86 [0.72,1.03]	0.82* [0.70,0.97]
Richer * KOF	0.80** [0.68,0.95]	0.78*** [0.68,0.90]	0.79* [0.66,0.96]	0.78** [0.67,0.91]
Richest * KOF	0.72*** [0.62,0.84]	0.73*** [0.64,0.83]	0.74*** [0.65,0.85]	0.70*** [0.61,0.82]
Interactions with Gross National Income				
GNI * KOF	0.99** [0.99,1.00]	0.99** [0.99,1.00]	0.99** [0.99,1.00]	0.99** [0.99,1.00]
Social KOF				
KOF	1.45*** [1.18,1.77]	1.33** [1.11,1.60]	1.63*** [1.32,2.00]	1.30* [1.05,1.61]
Interactions with wealth index quintiles				
Poorest * KOF	1.00 [1.00,1.00]	1.00 [1.00,1.00]	1.00 [1.00,1.00]	1.00 [1.00,1.00]
Poorer * KOF	1.04 [0.96,1.12]	1.08 [1.00,1.17]	1.04 [0.95,1.13]	1.06 [0.99,1.14]
Middle * KOF	1.02 [0.87,1.20]	1.11 [0.94,1.31]	1.02 [0.84,1.24]	1.07 [0.91,1.25]
Richer * KOF	1.06 [0.90,1.26]	1.13 [0.93,1.37]	1.06 [0.88,1.28]	1.10 [0.92,1.31]
Richest * KOF	1.06 [0.86,1.31]	1.14 [0.93,1.40]	1.06 [0.85,1.33]	1.11 [0.90,1.37]
Interactions with Gross National Income				
GNI * KOF	1.00 [0.99,1.00]	1.00 [0.99,1.01]	1.00* [0.99,1.00]	1.00 [0.99,1.01]
Political KOF				
KOF	0.90 [0.72,1.12]	0.82 [0.65,1.03]	0.87 [0.69,1.11]	0.85 [0.68,1.07]
Interactions with wealth index quintiles				
Poorest * KOF	1.00 [1.00,1.00]	1.00 [1.00,1.00]	1.00 [1.00,1.00]	1.00 [1.00,1.00]
Poorer * KOF	1.15*** [1.06,1.25]	1.12** [1.03,1.21]	1.14*** [1.06,1.22]	1.13** [1.04,1.24]
Middle * KOF	1.18 [0.98,1.42]	1.15 [0.93,1.42]	1.11 [0.93,1.33]	1.19 [0.96,1.47]
Richer * KOF	1.16 [0.91,1.49]	1.19 [0.92,1.55]	1.15 [0.89,1.47]	1.18 [0.90,1.54]
Richest * KOF	1.13 [0.81,1.59]	1.17 [0.82,1.65]	1.09 [0.77,1.53]	1.18 [0.83,1.68]
Interactions with Gross National Income				
GNI * KOF	1.00 [0.99,1.00]	1.00 [1.00,1.01]	1.00 [0.99,1.00]	1.01 [1.00,1.01]

* $p < 0.05$, ** $p < 0.01$, *** $p < 0.001$

Supplementary Table 8. Associations between the KOF Globalisation Index (per 10 units increase) and the double burden of malnutrition (stunted child with overweight mother) and their interactions with wealth index quintile and Gross National Income per capita, using 23 kg/m^2 as cut-off point for overweight for women in South Asian countries

	Economic KOF	Social KOF	Political KOF
KOF	1.51***	1.40***	0.86
	[1.22,1.87]	[1.17,1.67]	[0.69,1.08]
Interactions between KOF and wealth index quintiles			
Poorest * KOF (reference group)	1.00	1.00	1.00
	[1.00,1.00]	[1.00,1.00]	[1.00,1.00]
Poorer * KOF	0.96	1.06	1.13**
	[0.89,1.04]	[0.99,1.13]	[1.05,1.22]
Middle * KOF	0.84*	1.05	1.16
	[0.71,0.99]	[0.90,1.23]	[0.95,1.41]
Richer * KOF	0.79**	1.09	1.17
	[0.67,0.92]	[0.92,1.30]	[0.91,1.51]
Richest * KOF	0.72***	1.10	1.14
	[0.63,0.83]	[0.90,1.34]	[0.81,1.61]
Interaction with between KOF and Gross National Income (GNI)			
GNI * KOF	0.99**	1.00	1.00
	[0.99,1.00]	[0.99,1.01]	[1.00,1.01]

* p < 0.05, ** p < 0.01, *** p < 0.001

Supplementary Table 9. Associations between the KOF Globalisation Index (per 10 units increase) and the double burden of malnutrition (stunted child with overweight mother) and their interactions with wealth index quintile and Gross National Income per capita, weighted using normalised survey weights, i.e. all surveys are equally weighted within the sample

	Economic KOF	Social KOF	Political KOF
KOF	1.35***	1.49**	0.93
	[1.13,1.60]	[1.16,1.91]	[0.77,1.12]
Interactions between KOF and wealth index quintiles			
Poorest * KOF (reference group)	1.00	1.00	1.00
	[1.00,1.00]	[1.00,1.00]	[1.00,1.00]
Poorer * KOF	0.94	1.00	1.06
	[0.87,1.02]	[0.93,1.08]	[1.00,1.13]
Middle * KOF	0.83*	0.98	1.11
	[0.69,0.99]	[0.82,1.17]	[0.94,1.31]
Richer * KOF	0.75**	0.93	1.12
	[0.61,0.92]	[0.73,1.17]	[0.92,1.37]
Richest * KOF	0.68***	0.88	1.11
	[0.55,0.84]	[0.65,1.21]	[0.87,1.42]
Interaction with between KOF and Gross National Income (GNI)			
GNI * KOF	1.00**	0.99**	1.00
	[0.99,1.00]	[0.99,1.00]	[1.00,1.00]

* p < 0.05, ** p < 0.01, *** p < 0.001

Supplementary Table 10. Associations between the KOF Globalisation Index (per 10 units increase) and the double burden of malnutrition (stunted child with overweight mother) and their interactions with wealth index quintile and Gross National Income per capita at mother-level

	Economic KOF		*Social KOF*		*Political KOF*	
	Mother with at least one stunted child	Randomly selecting one child per household	Mother with at least one stunted child	Randomly selecting one child per household	Mother with at least one stunted child	Randomly selecting one child per household
KOF	1.50*** [1.22,1.85]	1.51*** [1.23,1.86]	1.42*** [1.20,1.69]	1.42*** [1.19,1.70]	0.88 [0.70,1.10]	0.86 [0.69,1.08]
Interactions with wealth index quintiles						
Poorest * KOF	1.00 [1.00,1.00]	1.00 [1.00,1.00]	1.00 [1.00,1.00]	1.00 [1.00,1.00]	1.00 [1.00,1.00]	1.00 [1.00,1.00]
Poorer * KOF	0.95 [0.88,1.03]	0.95 [0.88,1.02]	1.04 [0.97,1.12]	1.03 [0.97,1.11]	1.13** [1.04,1.23]	1.12* [1.02,1.21]
Middle * KOF	0.83* [0.71,0.98]	0.83* [0.71,0.98]	1.03 [0.88,1.21]	1.03 [0.90,1.20]	1.17 [0.95,1.43]	1.15 [0.94,1.41]
Richer * KOF	0.79** [0.67,0.92]	0.79** [0.68,0.92]	1.06 [0.90,1.26]	1.07 [0.91,1.25]	1.17 [0.91,1.52]	1.16 [0.90,1.50]
Richest * KOF	0.72*** [0.62,0.83]	0.72*** [0.62,0.83]	1.04 [0.85,1.27]	1.05 [0.87,1.27]	1.14 [0.81,1.62]	1.13 [0.81,1.59]
Interactions with Gross National Income						
GNI * KOF	0.99** [0.99,1.00]	0.99** [0.99,1.00]	1.00 [0.99,1.00]	1.00 [0.99,1.01]	1.00 [0.99,1.01]	1.00 [1.00,1.01]

* $p < 0.05$, ** $p < 0.01$, *** $p < 0.001$

Supplementary Table 11. Associations between the KOF Globalisation Index (per 10 units increase) and the double burden of malnutrition (stunted child with overweight mother) and their interactions with rural/urban area or mother's education and Gross National Income per capita

	Economic KOF		*Social KOF*		*Political KOF*	
	Rural/urban area	Mother education	Rural/urban area	Mother education	Rural/urban area	Mother education
KOF	1.34*** [1.15,1.57]	1.32*** [1.12,1.55]	1.42*** [1.21,1.67]	1.40*** [1.18,1.66]	1.04 [0.90,1.20]	1.00 [0.87,1.14]
Interactions with socioeconomic determinants						
Rural*KOF	1.00 [1.00,1.00]		1.00 [1.00,1.00]		1.00 [1.00,1.00]	
Urban*KOF	0.80*** [0.74,0.86]		0.95 [0.84,1.07]		1.01 [0.83,1.23]	
No mother education*KOF		1.00 [1.00,1.00]		1.00 [1.00,1.00]		1.00 [1.00,1.00]
Mother education*KOF		0.86* [0.75,0.99]		0.99 [0.90,1.10]		1.07 [0.93,1.23]
Interactions with Gross National Income						
GNI * KOF	1.00 [0.99,1.00]	0.99 [0.99,1.00]	1.00 [0.99,1.01]	1.00 [0.99,1.01]	1.00 [1.00,1.01]	1.00 [1.00,1.01]

* $p < 0.05$, ** $p < 0.01$, *** $p < 0.001$

Supplementary Table 12. Associations between the KOF Globalisation Index (per 10 units increase) and the double burden of malnutrition (stunted child with overweight mother) and their interactions with wealth index quintile and Gross National Income per capita, after adjusting for prevalence of child stunting and mother overweight by country, year, urban/rural region, and wealth index quintile.

	**Economic KOF**	**Social KOF**	**Political KOF**
KOF	1.57***	1.40**	0.93
	[1.30,1.90]	[1.13,1.74]	[0.69,1.25]
Interactions between KOF and wealth index quintiles			
Poorest * KOF	1.00	1.00	1.00
	[1.00,1.00]	[1.00,1.00]	[1.00,1.00]
Poorer * KOF	0.92*	1.04	1.11
	[0.86,0.99]	[0.96,1.13]	[0.99,1.25]
Middle * KOF	0.79***	1.03	1.12
	[0.70,0.89]	[0.86,1.23]	[0.86,1.46]
Richer * KOF	0.74***	1.03	1.10
	[0.66,0.84]	[0.81,1.31]	[0.78,1.56]
Richest * KOF	0.69***	1.02	1.07
	[0.59,0.80]	[0.77,1.36]	[0.69,1.66]
Interaction with between KOF and Gross National Income (GNI)			
GNI * KOF	0.99**	1.00	1.00
	[0.99,1.00]	[0.99,1.01]	[1.00,1.01]
Mother overweight	8.60**	4.93	5.35
	[2.28,32.41]	[0.80,30.42]	[0.78,36.89]
Standardised dominance statistic¥	29.43%	29.77%	29.86%
Child stunting	6.66*	4.86	4.63
	[1.45,30.68]	[0.43,54.39]	[0.50,43.30]
Standardised dominance statistic¥	8.55%	8.57%	8.59%

¥The standardised dominance statistic determines the contribution of the variable to the overall model fit

* p < 0.05, ** p < 0.01, *** p < 0.001
